# Biocontrol of Mycotoxin-Producing Fungi by Lactic Acid Bacteria

**DOI:** 10.3390/foods15111913

**Published:** 2026-05-28

**Authors:** Alice N. Mafe, Dietrich Büsselberg

**Affiliations:** 1 Department of Biological Sciences, Faculty of Sciences, Taraba State University, Main Campus, Jalingo 660101, Taraba State, Nigeria; mafealice1991@gmail.com; 2 Department of Physiology and Biophysics, Faculty of Medicine, Qatar Campus, Weill Cornell Medicine—Qatar, Education City, Qatar Foundation, Doha Metropolitan Area, Doha P.O. Box 22104, Qatar

**Keywords:** biocontrol microorganisms, microbial detoxification, antifungal metabolites, fermented food microbiota, toxin biotransformation, microbial food preservation, oxidative stress modulation, microbial safety strategies

## Abstract

Fungal contamination and the buildup of mycotoxins are ongoing threats to global food safety, especially in tropical areas where environmental conditions favor the growth of toxigenic fungi such as *Aspergillus* spp., *Fusarium* spp., and *Penicillium* spp. These toxins contaminate various food products and are linked to serious health problems, including liver toxicity, nerve toxicity, immune suppression, and cancer. Traditional methods to reduce these risks, such as chemical preservatives, heat treatments, and irradiation, have limited success in fully eliminating mycotoxins due to their stability, safety concerns, and declining consumer acceptance of synthetic additives. As a result, there is increasing interest in biological options that are safer and more sustainable. This review critically examines the potential of probiotic lactic acid bacteria (LAB) isolated from local fermented foods as multifunctional biocontrol agents that inhibit toxin-producing fungi, detoxify mycotoxins, and reduce cellular toxicity caused by these toxins. Scientific studies were retrieved from PubMed, ScienceDirect, Scopus, Web of Science, and Google Scholar, focusing on research published from 2011 to 2025 on antifungal activity, detoxification mechanisms, and cellular toxicology. The evidence shows that probiotic LAB employ various strategies, including producing organic acids, secreting bacteriocins, competing with fungi, adsorbing toxins onto their cell walls, and enzymatically transforming mycotoxins into less harmful substances. Recent findings also indicate that metabolites from LAB may influence oxidative stress, inflammation, and cell death in mammalian cells exposed to mycotoxins. Overall, probiotic LAB from native fermented foods offer promising biological approaches to improve food safety and reduce health risks associated with toxins. Future studies should focus on omics-based analysis of detoxification pathways, testing in real food systems, and translational research to support regulatory approval and large-scale use of probiotic-based strategies for mycotoxin control.

## 1. Introduction

### 1.1. Global Burden of Fungal Toxins in Food Systems

Fungal contamination and mycotoxin accumulation remain major global food safety challenges, as toxigenic fungi frequently contaminate crops during growth, processing, and storage. These heat-stable toxins persist in grains and cereal-based foods, posing serious risks to human and animal health due to their carcinogenic, mutagenic, teratogenic, immunosuppressive, and neurotoxic effects, while also causing substantial economic losses and threatening food security [1,2]. Mycotoxins are toxic secondary metabolites produced by several filamentous fungi, primarily *Aspergillus*, *Fusarium*, and *Penicillium* species, that frequently contaminate agricultural commodities during cultivation, harvest, storage, and processing. These toxins are widely detected in staple foods such as cereals, nuts, spices, and fermented products, making dietary exposure a major concern in both developed and developing countries [3,4]. The stability of many mycotoxins during food processing further exacerbates the problem, as conventional thermal treatments often fail to fully degrade these compounds [5,6,7]. Consequently, mycotoxin contamination has become a critical issue within global food supply chains, requiring continuous monitoring and effective mitigation strategies [8]. The health implications of chronic mycotoxin exposure are profound and multifaceted. Numerous mycotoxins have been linked to severe toxicological effects, including hepatotoxicity, nephrotoxicity, neurotoxicity, immunosuppression, and carcinogenesis [9,10]. For example, aflatoxin B1 is widely recognized as one of the most potent naturally occurring carcinogens, contributing significantly to the global burden of liver cancer [11,12]. In addition to direct human health effects, mycotoxins also pose significant risks to animal health, reducing productivity in livestock and poultry systems [13,14]. Beyond health impacts, mycotoxin contamination results in substantial economic losses worldwide through reduced crop value, trade restrictions, food waste, and increased costs associated with monitoring and regulatory compliance [15,16]. These challenges are particularly pronounced in tropical and subtropical regions, where warm temperatures and high humidity create ideal conditions for fungal proliferation and toxin production. As climate variability continues to influence fungal ecology, the risk of mycotoxin contamination in food systems is expected to increase, further emphasizing the need for innovative and sustainable control strategies.

### 1.2. Limitations of Conventional Mycotoxin Control Strategies

Several strategies have been developed to mitigate fungal contamination and reduce mycotoxin levels in food systems, including chemical preservatives, physical treatments, and post-harvest handling practices. Chemical preservatives and antifungal agents are widely used to inhibit fungal growth in stored foods; however, their use is increasingly limited due to concerns about toxicity, environmental impact, and the emergence of resistant fungal strains [17,18]. Moreover, consumer preferences are increasingly shifting toward minimally processed foods free from synthetic additives, thereby further restricting the use of chemical control methods [19,20,21].

Prevention of Fungal Growth: Preventive approaches are primarily designed to inhibit fungal proliferation before toxin formation occurs. These include post-harvest drying techniques that reduce water activity to levels unfavorable for fungal growth, as well as optimized storage conditions that limit moisture accumulation and oxygen availability [22]. Temperature control, particularly cold storage, is also widely employed to slow down fungal metabolism and delay spoilage processes [23]. In addition, the application of food-grade preservatives, including organic acids and salt-based compounds, contributes to suppressing fungal development during storage [24]. Physical interventions such as irradiation and controlled atmosphere storage further enhance fungal inhibition by disrupting spore viability and metabolic activity [25].

Detoxification and Removal of Existing Mycotoxins: Unlike preventive strategies, detoxification approaches aim to reduce or eliminate mycotoxins already present in contaminated food products. Chemical binders, such as clay minerals and activated carbon, are commonly used to adsorb mycotoxins and reduce their bioavailability in food and feed systems [3]. Ozonation has also been explored as an effective oxidative treatment capable of degrading certain mycotoxins through structural modification [26]. Similarly, ammoniation is applied in some feed systems to chemically alter toxin structures, thereby reducing their toxicity [27]. Enzymatic degradation is a more targeted approach in which specific enzymes catalyze the breakdown of mycotoxins into less harmful metabolites [28]. Despite these advances, the efficiency of detoxification methods varies significantly depending on the type of toxin, food matrix, and processing conditions.

Physical sorting and cleaning techniques may remove visibly contaminated materials; however, they often fail to eliminate hidden or subclinical contamination, limiting their effectiveness as standalone interventions [29]. Likewise, many mycotoxins are chemically stable and resistant to conventional processing conditions, further constraining the overall efficiency of detoxification strategies. These limitations highlight the need for alternative strategies capable not only of inhibiting fungal growth but also of detoxifying or transforming mycotoxins into less harmful compounds. LAB-based biocontrol should therefore be viewed as a complementary strategy within integrated mycotoxin management systems rather than a complete replacement for conventional approaches.

### 1.3. Indigenous Fermented Foods as Reservoirs of Functional Microbiota

Traditional fermented foods represent complex microbial ecosystems that harbor diverse communities of beneficial microorganisms with functional and technological properties [30,31]. In many African countries, particularly Nigeria, a wide variety of fermented foods such as ogi, garri, iru, fufu, and zobo, are produced through spontaneous fermentation driven by naturally occurring microbiota. These fermentation systems create unique ecological environments where microorganisms compete for nutrients and survival, leading to the selection of microbial strains with strong antimicrobial and metabolic capabilities [32,33]. Among the microorganisms associated with fermented foods, lactic acid bacteria (LAB) are particularly prominent due to their ability to produce organic acids, antimicrobial peptides, and other bioactive metabolites that inhibit spoilage and pathogenic organisms [34,35]. The metabolic versatility of LAB enables them to adapt to diverse environmental conditions and exert antagonistic effects against competing microbes, including toxigenic fungi [36,37]. Furthermore, the long history of safe consumption of fermented foods has established many LAB species as generally recognized as safe (GRAS), making them attractive candidates for application in food preservation and safety interventions [38,39,40]. Despite the rich microbial diversity in indigenous fermented foods, the potential of these ecosystems as reservoirs of antifungal and detoxifying microorganisms remains underexplored, particularly for mycotoxin control.

### 1.4. Emerging Interest in Probiotic-Based Biocontrol

In recent years, increasing attention has been paid to the use of probiotic microorganisms as biological control agents in food systems [41,42]. Probiotic LAB are known to produce a wide range of antimicrobial metabolites, including organic acids, hydrogen peroxide, bacteriocins, and other bioactive compounds that can inhibit the growth of fungal pathogens [43,44,45]. These metabolites alter the microenvironment by lowering pH, disrupting fungal cell membranes, and interfering with fungal metabolic processes, thereby limiting the proliferation of mycotoxin-producing fungi [46,47]. Beyond growth inhibition, probiotic LAB have also demonstrated the capacity to interact directly with mycotoxins through adsorption onto bacterial cell-wall components or enzymatic transformation into less-toxic derivatives [48,49]. Such detoxification mechanisms highlight the potential of LAB not only to prevent fungal contamination but also to reduce toxin levels in contaminated foods [50]. The application of probiotic-based biocontrol strategies is therefore gaining traction as a natural and sustainable alternative to chemical preservatives. Additionally, integrating probiotic cultures into food fermentation and preservation processes offers the dual advantage of improving food safety and enhancing nutritional and functional properties.

### 1.5. Toxicological Effects of Mycotoxins on Human Cells

The toxicological effects of mycotoxins extend beyond acute poisoning and include a wide range of cellular and molecular disruptions that contribute to chronic diseases [51,52]. At the cellular level, many mycotoxins exert their toxicity by inducing oxidative stress, characterized by excessive production of reactive oxygen species (ROS) and subsequent damage to cellular macromolecules, including lipids, proteins, and DNA [53,54]. Oxidative stress plays a central role in mediating cytotoxicity, characterized by excessive production of reactive oxygen species (ROS) and subsequent damage to cellular macromolecules, resulting in toxic and genotoxic effects of several mycotoxins, including aflatoxins and ochratoxins [55,56]. In addition to oxidative damage, mycotoxins have been shown to disrupt mitochondrial function, impair energy metabolism, and trigger programmed cell death pathways [57,58]. Mitochondrial dysfunction often activates apoptotic signaling cascades involving key regulatory proteins such as caspases and Bcl-2 family members [59,60]. These molecular events contribute to tissue damage and have been implicated in the development of cancer, neurodegenerative disorders, and immune dysfunction. Emerging evidence also suggests that certain mycotoxins can interfere with neuronal signaling pathways, highlighting potential links between dietary toxin exposure and neurotoxicity. Understanding these cellular mechanisms is essential for developing strategies that not only reduce mycotoxin contamination in foods but also mitigate their biological impacts on human health.

### 1.6. Knowledge Gaps in Current Research

Despite substantial advances in the study of mycotoxins, significant gaps remain in understanding their biological mitigation, particularly through microbial interventions. While current literature has extensively addressed mycotoxin occurrence, detection, and regulatory thresholds, comparatively less emphasis has been placed on elucidating the functional and mechanistic basis of microbial detoxification. In particular, the molecular pathways through which lactic acid bacteria (LAB) and other beneficial microorganisms bind, degrade, or transform mycotoxins remain incompletely characterized. This limited mechanistic resolution constrains the rational development of reproducible and scalable biocontrol strategies for food safety applications [61,62]. Four principal research gaps are identified.

Strain-dependent variability: A critical and consistently reported limitation is the pronounced strain-level heterogeneity in LAB functionality. Even within the same species, different strains exhibit substantial variation in antifungal activity, adsorption capacity, and enzymatic detoxification potential [63]. However, a considerable proportion of studies report outcomes at the species level without adequate genomic or phenotypic strain resolution [64,65]. This undermines reproducibility and limits the ability to identify robust, high-performance strains suitable for standardized applications in mycotoxin control.

Limited validation in real food matrices: Most experimental evidence for LAB-mediated antifungal and detoxification activity comes from in vitro assays conducted under simplified laboratory conditions. Such systems fail to capture the physicochemical complexity of real food matrices, including variations in water activity, pH, nutrient composition, microbial competition, and processing-induced stressors [66,67]. Consequently, there is insufficient translational evidence demonstrating the consistent efficacy of LAB-based interventions in real food systems under storage, processing, or supply chain conditions.

Toxicological uncertainty of transformation products: Although microbial biotransformation of mycotoxins is widely reported, the toxicological safety of resulting metabolites remains inadequately established [68]. In several cases, degradation or transformation does not equate to detoxification, as intermediate or end-products may retain residual toxicity or exhibit altered biological activity. This presents a critical safety concern, underscoring the need for integrated toxicological evaluation, including in vitro and in vivo validation of metabolite safety profiles, alongside biochemical characterization.

Regulatory and commercialization barriers: Despite promising laboratory-scale findings, the translation of LAB-based detoxification strategies into commercially viable applications remains limited. This is largely due to the absence of harmonized regulatory frameworks governing microbial detoxification agents, insufficient safety validation, including whole-genome sequencing and virulence profiling, and a lack of standardized efficacy benchmarks for industrial approval [49]. These regulatory and standardization gaps significantly impede the commercialization and large-scale deployment of microbial biocontrol technologies in food systems.

Additionally, the microbial diversity of traditional and African fermented foods remains underexplored in global datasets, despite their potential as reservoirs of novel LAB strains with unique antifungal and detoxification capabilities [69]. Addressing these gaps requires an integrated, multidisciplinary approach that combines microbial genomics, food matrix science, toxicology, and regulatory harmonization to enable the reliable and scalable application of LAB-based mycotoxin control strategies.

### 1.7. Scope and Aim of the Review

Given the growing need for sustainable and effective strategies to control mycotoxin contamination, there is increasing interest in exploring the potential of probiotic microorganisms derived from traditional fermented foods. This review critically evaluates probiotic lactic acid bacteria isolated from indigenous fermented foods as multifunctional agents for controlling mycotoxin-producing fungi. Particular emphasis is placed on elucidating the microbial antifungal mechanisms, enzymatic detoxification pathways, and potential protective effects of probiotic metabolites against mycotoxin-induced cellular toxicity. By integrating insights from food microbiology, biochemical detoxification processes, and cellular toxicology, this review aims to provide a comprehensive perspective on the emerging role of probiotic LAB in enhancing food safety and mitigating the health risks associated with fungal toxins.

## 2. Methodology

### 2.1. Literature Search Strategy

The scientific literature used for this narrative review was systematically retrieved from several widely recognized academic databases, including PubMed, Scopus, ScienceDirect, Web of Science, and Google Scholar. These databases were selected because they provide extensive coverage of peer-reviewed publications across disciplines relevant to food microbiology, toxicology, biotechnology, and food safety. The search focused on studies investigating the antifungal activity of probiotic microorganisms, microbial detoxification of mycotoxins, and the biological mechanisms underlying toxin mitigation in food systems. The literature search was conducted to capture both foundational and recent developments in the field, ensuring a comprehensive representation of current knowledge and emerging research trends. To capture relevant publications, a combination of specific and related search terms was employed during the database queries. The primary keywords used in the literature search included “lactic acid bacteria,” “mycotoxin detoxification,” “antifungal activity,” “probiotic biocontrol,” “fermented foods,” and “mycotoxin toxicity.” Additional related search phrases included “LAB antifungal activity,” “probiotic detoxification of mycotoxins,” “microbial biocontrol fungi,” “fermented foods microbiota,” “aflatoxin degradation bacteria,” and “microbial toxin detoxification mechanisms.” These keywords were used individually and in combination using Boolean operators such as AND and OR to broaden the search scope and identify studies addressing microbial inhibition of toxigenic fungi, enzymatic detoxification pathways, and probiotic-mediated strategies to enhance food safety. The screening process involved multiple stages, including title screening, abstract screening, and full-text review. This approach ensured that only studies directly relevant to the review’s objectives were retained for detailed evaluation.

### 2.2. Inclusion and Exclusion Criteria

To ensure the scientific relevance and quality of the reviewed literature, clear inclusion and exclusion criteria were applied during the selection process. The inclusion criteria focused on peer-reviewed English-language articles published between 2011 and 2025, covering topics related to lactic acid bacteria (LAB), antifungal activity, microbial detoxification of mycotoxins, probiotic metabolites, and the toxicological effects of mycotoxins on biological systems. Only peer-reviewed articles were considered eligible for inclusion. Primary emphasis was placed on original experimental research articles, while selected review papers were used only for background information and contextual interpretation. Relevant original research papers and selected book chapters that provided mechanistic insights or significant experimental findings were also considered. Studies were excluded if they were not written in English, were not peer-reviewed, or lacked sufficient scientific relevance to fungal toxin mitigation. Publications that focused solely on unrelated microbial processes, non-food systems, or lacked methodological clarity were also omitted to maintain the rigor and focus of the review. Additional exclusion criteria included conference abstracts, duplicated studies, non-English articles, and papers lacking mechanistic relevance to microbial detoxification or antifungal activity.

### 2.3. Data Extraction and Synthesis

The initial database search yielded approximately 1500 publications on microbial antifungal activity, mycotoxin detoxification, and probiotic applications in food systems. Duplicate records were identified and removed using Mendeley Desktop reference management software to ensure the dataset’s accuracy and integrity. Following this, the remaining publications underwent a systematic screening process in which titles and abstracts were evaluated for relevance to the review’s objectives. Articles that met the preliminary criteria were subsequently subjected to full-text evaluation to assess their methodological quality, relevance, and contribution to the review topic. After this rigorous selection process, approximately 180 studies were retained for the final analysis of this review. Data extracted from the selected studies included the LAB species investigated, food source or substrate, target fungal species, detoxification or antifungal mechanisms, percentage reductions in mycotoxins or fungal growth, and observed cellular or toxicological outcomes. The extracted information was qualitatively synthesized to identify common trends, mechanistic pathways, and emerging applications of probiotic-mediated mycotoxin detoxification and fungal biocontrol in food systems.

## 3. Ecology of Mycotoxin-Producing Fungi in Food Systems

### 3.1. Major Genera and Environmental Factors

#### 3.1.1. Major Genera

Mycotoxin contamination in food systems is predominantly associated with filamentous fungi in the genera *Aspergillus*, *Fusarium*, and *Penicillium*. These fungi are widely distributed in soil, plant materials, and agricultural environments, enabling them to colonize crops both before and after harvest [70]. Their ecological adaptability enables them to thrive in diverse environmental conditions, making them persistent contaminants of food commodities such as cereals, nuts, spices, and processed foods [71]. The ability of these fungi to produce toxic secondary metabolites during growth or storage poses significant risks to food safety and human health. Species within the genus *Aspergillus* spp. are among the most significant producers of mycotoxins, particularly in tropical and subtropical regions [72,73]. Several species, including *Aspergillus flavus* and *Aspergillus parasiticus*, are well known for producing aflatoxins, which are highly carcinogenic compounds frequently detected in maize, groundnuts, tree nuts, and other oilseeds [74,75]. These fungi thrive in warm and humid conditions and often contaminate crops during both field growth and post-harvest storage [76,77]. Due to the stability of aflatoxins during processing, contamination by *Aspergillus* species remains one of the most serious food safety concerns worldwide. The genus *Fusarium* is another important group of toxigenic fungi commonly associated with cereal crops such as maize, wheat, and barley [78]. *Fusarium* species are particularly problematic because they infect crops during the pre-harvest stage and can persist through processing and storage. These fungi produce several classes of mycotoxins, including fumonisins, trichothecenes, and zearalenone, which have been linked to various toxicological effects such as immunosuppression, reproductive disorders, and carcinogenicity [79,80]. Their widespread presence in staple crops contributes significantly to global dietary exposure to mycotoxins. Similarly, species belonging to the genus *Penicillium* are important contributors to mycotoxin contamination, particularly during the storage of food commodities. These fungi are commonly found in temperate climates and produce toxins such as ochratoxin A and patulin [81,82]. *Penicillium* species frequently colonize stored grains, fruits, and processed foods under poor storage conditions [83]. For instance, patulin contamination is often associated with spoiled apples and fruit-based products, whereas ochratoxin A has been detected in cereals, coffee, and dried fruits. The ability of *Penicillium* species to grow at relatively low temperatures enables them to proliferate even in refrigerated storage.

#### 3.1.2. Environmental Factors Influencing Fungal Growth and Toxin Production

The occurrence and severity of mycotoxin contamination in food systems are strongly influenced by environmental conditions that favor fungal growth and toxin biosynthesis [84]. Among these factors, humidity plays a critical role in determining fungal proliferation and metabolic activity [85]. High moisture levels create favorable conditions for fungal colonization of crops and stored food products [86]. Water activity (_a_w) above critical thresholds enables toxigenic fungi to grow and produce mycotoxins, particularly in poorly dried or inadequately stored commodities [87]. In tropical regions where relative humidity remains consistently high, the risk of fungal contamination and toxin accumulation is significantly increased. Storage conditions also represent a major determinant of fungal contamination in food systems. Improper storage practices, such as inadequate ventilation, high temperatures, and prolonged storage, can promote the growth of toxigenic fungi in harvested crops [88]. Post-harvest contamination is particularly common in developing regions where storage infrastructure may be limited. In such environments, grains and other food materials are often stored under conditions that promote fungal growth, leading to increased mycotoxin accumulation over time [89]. Effective storage management, including proper drying and controlled environmental conditions, is therefore essential for reducing fungal proliferation and toxin production. Another important factor influencing fungal ecology in food systems is substrate composition. Different food matrices provide distinct nutrient profiles that affect fungal growth and toxin biosynthesis [90]. Crops rich in carbohydrates and lipids, such as maize, peanuts, and other cereals, are particularly susceptible to colonization by toxigenic fungi [91]. Nutrient availability, pH, and the presence of competing microorganisms within the food matrix can also influence fungal metabolism and toxin production [92]. These interactions highlight the complexity of fungal ecology in food systems and underscore the need for integrated strategies that address both environmental and biological determinants of mycotoxin contamination. A comprehensive overview of the most important mycotoxins occurring in food systems is provided in Table 1.

### 3.2. Indigenous Fermented Foods as Sources of Antifungal Lactic Acid Bacteria

#### 3.2.1. Indigenous Fermented Foods as Microbial Reservoirs

Traditional fermented foods represent complex microbial ecosystems that harbor a wide range of beneficial microorganisms with important technological and functional properties [99]. In many African countries, particularly Nigeria, fermentation remains a widely practiced method for food preservation and enhancement of nutritional quality. These fermentation processes are often spontaneous and rely on naturally occurring microbiota present in the raw materials and the surrounding environment [100]. As a result, indigenous fermented foods serve as rich reservoirs of diverse microbial populations, particularly lactic acid bacteria (LAB), which play central roles in fermentation, food safety, and microbial stability [101]. Among the commonly consumed Nigerian fermented foods, ogi is a cereal-based fermented product prepared from maize, sorghum, or millet and is widely consumed as a breakfast meal or weaning food [102]. During fermentation, LAB dominate the microbial community and contribute to acidification, flavor development, and the inhibition of spoilage organisms [103]. Several studies have identified LAB species isolated from ogi with strong antimicrobial and antifungal properties, suggesting that this fermented product is a valuable source of bioactive microbial strains with potential applications in food preservation and mycotoxin control [104]. Another widely consumed beverage is zobo, a traditional drink produced from the calyces of *Hibiscus sabdariffa*. Although zobo preparation often involves boiling the plant material, microbial contamination can occur during post-processing handling and storage. Fermentation-associated LAB isolated from zobo and related plant-based beverages have been shown to produce organic acids and antimicrobial compounds that inhibit spoilage microorganisms and toxigenic fungi [105]. These properties highlight the potential of LAB derived from zobo fermentation environments to serve as natural biocontrol agents in food systems.

Iru is a fermented condiment produced from African locust beans (*Parkia biglobosa*), which also hosts a diverse microbial community during fermentation. While *Bacillus* species dominate the fermentation process, LAB are frequently detected as secondary microbiota and contribute to microbial interactions within the fermenting substrate [106,107]. These LAB strains may produce antimicrobial metabolites that influence microbial succession and inhibit competing organisms, including fungal contaminants. Similarly, garri, a fermented cassava product widely consumed across West Africa, undergoes fermentation that promotes the growth of LAB capable of producing antimicrobial metabolites. The acidic environment generated during fermentation suppresses pathogenic microorganisms and improves the safety and shelf-life of the final product [108,109]. LAB isolated from garri fermentation systems has demonstrated inhibitory effects against several fungal species, indicating their potential utility in controlling mycotoxin-producing fungi [110]. Another important staple food is fufu, a fermented cassava dough that undergoes natural fermentation before consumption. The fermentation process encourages the proliferation of LAB species that contribute to acidification and microbial stabilization of the food matrix. These microorganisms not only enhance food preservation but may also produce metabolites capable of inhibiting spoilage fungi and reducing contamination risks [111,112]. Collectively, these indigenous fermented foods provide a rich and largely untapped reservoir of LAB strains with significant antifungal and biocontrol potential.

#### 3.2.2. Mechanisms of Microbial Competition in Fermented Food Ecosystems

Microbial competition plays a crucial role in shaping the microbial ecology of fermented foods and significantly suppresses undesirable microorganisms, including toxigenic fungi [113]. Within fermentation environments, LAB establish dominance through rapid growth, efficient nutrient utilization, and the production of metabolites that inhibit competing microbial species [114]. One of the most important mechanisms of microbial competition is the acidification of the fermentation environment, primarily through the production of lactic acid and other organic acids [115]. These acids lower the pH of the food matrix, creating conditions unfavorable to the growth of many fungal contaminants. In addition to acid production, LAB synthesize a variety of antimicrobial compounds that contribute to their competitive advantage. These include bacteriocins, hydrogen peroxide, diacetyl, and other secondary metabolites that disrupt the cellular integrity and metabolic activity of competing microorganisms [116,117]. Some LAB strains also produce volatile organic compounds with antifungal properties, further enhancing their ability to inhibit fungal growth in food matrices [118]. Another important mechanism involves competition for essential nutrients and ecological niches. During fermentation, LAB rapidly consume available carbohydrates and other nutrients required for microbial growth, thereby limiting the resources available to competing organisms such as toxigenic fungi [119,120]. This competitive exclusion reduces the likelihood of fungal colonization and subsequent mycotoxin production in fermented food systems. Furthermore, certain LAB strains can interact directly with fungal cells, either by producing cell wall-degrading enzymes or by forming biofilms that limit fungal attachment and growth [121,122]. These multifaceted mechanisms of microbial competition underscore the importance of LAB in maintaining microbial balance in fermented foods and highlight their potential as natural biocontrol agents for food preservation and mycotoxin mitigation. Representative lactic acid bacteria isolated from fermented foods that exhibit antifungal activity are summarized in Table 2.

### 3.3. Mechanisms of Antifungal Activity by Probiotic Lactic Acid Bacteria

#### 3.3.1. Organic Acid Production

One of the primary mechanisms through which probiotic lactic acid bacteria (LAB) inhibit fungal growth is the production of organic acids during carbohydrate fermentation [129]. LAB metabolize available sugars to produce metabolites such as lactic acid and acetic acid, which significantly reduce the pH of the surrounding environment. This acidification creates unfavorable conditions for the growth and proliferation of many spoilage and toxigenic fungi [110]. The inhibitory effect of organic acids is not solely due to reduced pH, as the undissociated acid molecules can penetrate fungal cell membranes and disrupt intracellular metabolic processes [130]. Once inside the fungal cell, these acids dissociate and release protons, leading to cytoplasmic acidification, disruption of enzymatic activity, and impairment of energy metabolism [131]. Lactic acid is the most abundant organic acid produced by LAB during fermentation and plays a critical role in suppressing fungal colonization in food matrices. By lowering the pH to levels below the optimal range for fungal growth, lactic acid inhibits the germination of fungal spores and slows the expansion of fungal hyphae [132]. Additionally, lactic acid can interfere with fungal membrane integrity and alter nutrient transport systems, further contributing to antifungal activity [133]. Acetic acid, although typically produced in smaller quantities, possesses stronger antifungal properties due to its higher lipophilicity and ability to penetrate microbial membranes more effectively [134]. The synergistic action of lactic and acetic acids, therefore, contributes significantly to the overall antifungal capacity of LAB in fermented foods and preservation systems.

#### 3.3.2. Bacteriocins

In addition to organic acid production, many LAB strains produce bacteriocins, which are ribosomally synthesized antimicrobial peptides that inhibit a wide range of microorganisms [135]. Although bacteriocins are primarily known for their antibacterial properties, several studies have demonstrated that certain bacteriocins also exhibit inhibitory effects against fungi and yeasts. These peptides function by disrupting cell membrane integrity, forming pores in microbial membranes, or interfering with essential cellular processes such as DNA replication and protein synthesis [136]. Some bacteriocins, such as nisin and pediocin, have been shown to affect fungal cells, either directly or indirectly, by weakening cell membranes and increasing fungal susceptibility to other antimicrobial metabolites [137]. The production of bacteriocins provides LAB with a competitive advantage in microbial ecosystems, allowing them to suppress the growth of competing microorganisms, including toxigenic fungi associated with food spoilage and mycotoxin production [138]. In fermented food systems, bacteriocin-producing LAB contribute to microbial stability and enhance the safety and shelf-life of food products [139].

#### 3.3.3. Competitive Nutrient Exclusion

Another important mechanism through which probiotic LAB exert antifungal effects is competitive nutrient exclusion. Within complex microbial ecosystems such as fermented foods, microorganisms compete for essential nutrients, ecological niches, and metabolic resources. LAB possess rapid growth rates and efficient carbohydrate utilization pathways, allowing them to dominate fermentation environments and consume nutrients required by competing microorganisms [140]. By rapidly metabolizing available sugars, amino acids, and micronutrients, LAB limit the resources available to fungal species attempting to colonize the same environment. This competitive advantage restricts fungal growth and reduces the likelihood of toxin production [141]. Furthermore, the formation of dense LAB populations within the food matrix can physically limit fungal colonization by occupying available ecological niches and preventing fungal spores from establishing growth [142]. The combined effects of nutrient depletion and microbial dominance significantly suppress toxigenic fungi in fermented food systems.

#### 3.3.4. Volatile Antifungal Compounds

**Figure 1:**
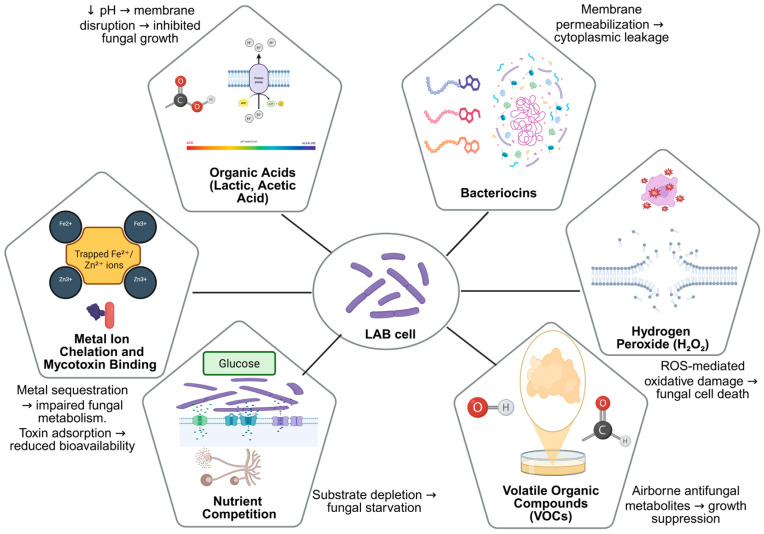
Mechanisms of LAB-mediated inhibition of mycotoxin-producing fungi (Created in BioRender. Dietrich B. (2026)).

In addition to soluble metabolites, probiotic LAB are capable of producing volatile organic compounds (VOCs) that exhibit antifungal properties. These volatile compounds can diffuse through the surrounding environment and inhibit fungal growth without direct contact between microorganisms [143,144]. Examples of antifungal VOCs produced by LAB include diacetyl, acetoin, ethanol, and various organic aldehydes and ketones [118]. These compounds interfere with fungal metabolic processes, disrupt membrane structures, and inhibit spore germination. Volatile antifungal compounds are particularly important in food preservation systems where microbial interactions occur within confined environments such as storage containers and packaging. Because VOCs can spread through the headspace of food matrices, they provide an additional layer of protection against fungal contamination [145]. The production of such compounds complements other antifungal mechanisms of LAB, including acidification and bacteriocin production, resulting in a multifaceted antimicrobial strategy [146]. Collectively, these mechanisms highlight the significant potential of probiotic LAB as natural biocontrol agents that inhibit mycotoxin-producing fungi and improve the microbiological safety of food products. The mechanisms by which probiotic lactic acid bacteria inhibit the growth of mycotoxin-producing fungi are illustrated in Figure 1.

### 3.4. Enzymatic and Biochemical Pathways for Mycotoxin Detoxification

#### 3.4.1. Enzymatic Degradation

Enzymatic degradation represents one of the most effective biological strategies for the detoxification of mycotoxins in food systems [147]. Certain probiotic lactic acid bacteria (LAB) produce enzymes that transform complex mycotoxin molecules into less toxic or non-toxic compounds through biochemical modification. These enzymatic reactions alter the structural integrity of mycotoxins, thereby reducing their biological activity and toxicity. Such mechanisms are particularly important in fermented food matrices where LAB are metabolically active and capable of producing various detoxifying enzymes [148]. Among the key enzymes implicated in microbial mycotoxin degradation are esterases, oxidoreductases, and lactonases. Esterases catalyze the hydrolysis of ester bonds in certain mycotoxins, thereby breaking down their molecular structure and reducing toxicity [149]. Oxidoreductases catalyze redox reactions that modify functional groups within mycotoxin molecules, often resulting in structural rearrangements that reduce their biological activity [150]. These enzymes can either oxidize or reduce specific chemical groups within the toxin structure, producing metabolites that are less harmful to humans and animals. Lactonases also play a crucial role in detoxification by targeting lactone rings essential to the toxicity of several mycotoxins. The enzymatic opening of these lactone rings disrupts the toxin’s active conformation, rendering it biologically inactive or significantly less toxic [151]. Through these enzymatic activities, LAB contribute to the biodegradation of mycotoxins during fermentation and storage, providing a natural and environmentally friendly approach to reducing toxin levels in contaminated foods.

#### 3.4.2. Cell Wall Binding Mechanisms

In addition to enzymatic degradation, LAB can reduce mycotoxin levels through cell-wall binding mechanisms. This process involves the physical adsorption of mycotoxin molecules onto structural components of the bacterial cell wall, thereby preventing their absorption in the gastrointestinal tract or their persistence in food systems. The binding process is largely attributed to the complex architecture of the LAB cell wall, which contains macromolecules such as peptidoglycan, teichoic acids, polysaccharides, and surface proteins [152]. Among these components, peptidoglycan plays a significant role in mycotoxin adsorption. The porous and highly cross-linked structure of peptidoglycan provides multiple binding sites that interact with toxin molecules via hydrogen bonding, hydrophobic interactions, and electrostatic forces. This adsorption process effectively immobilizes mycotoxins on the bacterial surface, thereby reducing their bioavailability and toxicity. Although this mechanism does not chemically degrade the toxin, it significantly contributes to detoxification by sequestering harmful compounds and preventing their interaction with host tissues [153]. Furthermore, toxin-binding efficiency may vary with factors such as bacterial strain, cell wall composition, environmental pH, and the structural characteristics of the mycotoxin involved. Both viable and non-viable LAB cells have been shown to exhibit binding capabilities, suggesting that cell wall architecture rather than metabolic activity primarily drives this detoxification pathway [154]. As a result, LAB cell-wall binding has gained attention as a promising strategy to reduce mycotoxin contamination in food and feed systems.

#### 3.4.3. Metabolite Transformation Pathways

**Figure 2:**
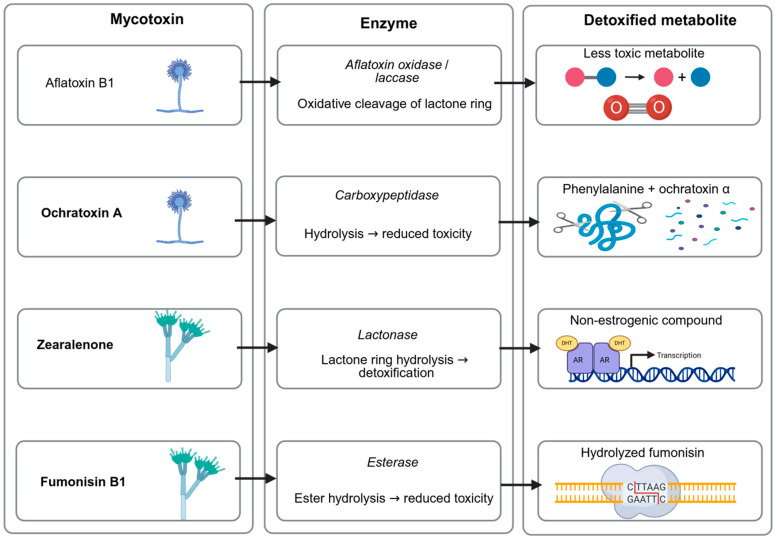
Enzymatic pathways of microbial mycotoxin detoxification (Created in BioRender. Dietrich B. (2026)).

Another important pathway for mycotoxin detoxification involves metabolite transformation, in which microbial metabolism converts toxic compounds into structurally modified derivatives with reduced toxicity. These transformations occur through various biochemical reactions, including hydroxylation, reduction, hydrolysis, and demethylation, which may reduce the toxicity of the parent mycotoxins [155]. LAB and other microorganisms can transform mycotoxins into metabolites that exhibit lower biological activity. These metabolic transformations may occur during fermentation or through enzymatic reactions facilitated by microbial enzymes [156]. A notable example is the transformation of aflatoxin B1, one of the most potent and carcinogenic mycotoxins, into less toxic derivatives. Certain LAB strains can modify aflatoxin B1 through enzymatic or metabolic processes that alter its reactive sites and reduce its ability to bind to cellular macromolecules, such as DNA and proteins [157]. These modifications can lead to the formation of compounds with significantly reduced mutagenic and carcinogenic potential. The efficiency of metabolite transformation depends on several factors, including the microbial strain involved, environmental conditions, and the chemical structure of the mycotoxin. In many cases, the detoxification process involves a combination of enzymatic degradation, adsorption, and metabolic conversion [158]. Together, these biochemical pathways highlight the potential of LAB as natural biocontrol agents that can mitigate mycotoxin contamination in food systems while maintaining the nutritional and sensory quality of fermented products. The biochemical and enzymatic pathways involved in microbial mycotoxin detoxification are summarized in Figure 2. The different microbial mechanisms involved in mycotoxin detoxification are outlined in Table 3.

### 3.5. Probiotic LAB in Food Preservation Systems

Probiotic lactic acid bacteria (LAB) have gained significant attention as natural biopreservatives in food systems due to their ability to inhibit spoilage microorganisms, suppress mycotoxin-producing fungi, and improve the microbiological safety of food products [165]. Their role in food preservation is largely attributed to the production of antimicrobial metabolites, competitive interactions with microbes, and detoxification mechanisms that limit fungal growth and toxin accumulation [166]. As consumers increasingly demand minimally processed foods with fewer chemical preservatives, LAB-based preservation strategies have emerged as promising alternatives in both traditional and industrial food systems. These bacteria are particularly valuable in fermented foods, where they contribute not only to product stability but also to enhanced nutritional and functional qualities [167]. Several studies have demonstrated the application of probiotic LAB in diverse food matrices, including plant-based beverages, cereal-based fermentations, and dairy alternatives [168,169,170]. In these systems, LAB can inhibit the growth of toxigenic fungi such as *Aspergillus* spp., *Penicillium* spp., and *Fusarium* spp., thereby reducing the risk of mycotoxin contamination. Additionally, their metabolic activities help maintain favorable storage conditions by lowering pH, producing antimicrobial compounds, and stabilizing microbial ecosystems within the food matrix [171]. As a result, LAB are increasingly being explored as multifunctional agents capable of improving both food preservation and safety.

#### 3.5.1. Zobo Preservation

Zobo, a traditional beverage made from the calyces of *Hibiscus sabdariffa*, is widely consumed across many parts of Africa for its high moisture content and nutrient availability. Incorporating probiotic LAB into Zobo fermentation or storage systems has been shown to enhance its microbial stability and extend shelf life. LAB strains, such as *Lactobacillus plantarum*, can inhibit spoilage microorganisms and reduce fungal contamination through acidification and the production of antimicrobial metabolites. These activities help maintain the sensory quality of the beverage while reducing the potential for mycotoxin contamination during storage [172].

#### 3.5.2. Fermented Beverages

Fermented beverages represent another important category where LAB play a significant role in food preservation. Traditional and modern fermented drinks often rely on LAB-driven fermentation processes to improve flavor, stability, and safety [173]. During fermentation, LAB produce organic acids, bacteriocins, and volatile compounds that inhibit the growth of pathogenic and spoilage microorganisms. In addition to enhancing shelf stability, these metabolites can suppress mycotoxin-producing fungi that may contaminate raw materials or storage environments [174]. The use of selected probiotic LAB strains in fermented beverage production, therefore, offers a dual benefit: improving the functional properties of the beverage while reducing microbial hazards [175].

#### 3.5.3. Dairy Analogues

With the increasing demand for plant-based diets, dairy analogues derived from soy, almond, coconut, and other plant sources have become popular alternatives to traditional dairy products. However, plant-based raw materials can be susceptible to fungal contamination and mycotoxin formation during processing and storage. The incorporation of probiotic LAB in dairy analogue fermentation has been shown to enhance microbial safety by inhibiting fungal growth and detoxifying certain mycotoxins [176]. LAB fermentation also improves the sensory and nutritional properties of these products by enhancing protein digestibility, producing bioactive compounds, and contributing desirable flavors and textures. Consequently, LAB are increasingly being utilized as natural preservation agents in the development of stable and safe dairy alternative products [177].

#### 3.5.4. Cereal Fermentations

Cereal-based fermented foods are widely consumed in many regions of the world and serve as important sources of nutrients and dietary energy. However, cereals are particularly vulnerable to contamination by mycotoxin-producing fungi during cultivation, harvesting, and storage [178]. Fermentation with probiotic LAB has been shown to reduce fungal growth and lower mycotoxin levels in cereal-based foods. LAB fermentation improves the safety of cereals by producing organic acids that inhibit fungal proliferation and by promoting detoxification mechanisms that reduce toxin bioavailability [179]. Additionally, LAB contribute to the improvement of nutritional quality by enhancing mineral bioavailability, degrading antinutritional factors, and generating beneficial metabolites [180]. These characteristics highlight the important role of LAB in improving the safety and stability of cereal fermentation systems. Practical applications of LAB for controlling mycotoxin contamination in food systems are presented in Table 4.

### 3.6. Cellular and Molecular Impacts of Mycotoxins

Mycotoxins are toxic secondary metabolites produced by certain fungal species that can exert profound effects on cellular and molecular processes in humans and animals. Exposure to these toxins through contaminated food can disrupt multiple biological systems and contribute to the development of acute and chronic health conditions [187]. At the cellular level, mycotoxins interfere with metabolic pathways, damage cellular organelles, and disrupt signaling processes that regulate cell survival and immune responses. These toxic effects are often mediated through mechanisms such as oxidative stress, mitochondrial damage, apoptosis, and inflammatory signaling pathways [188].

#### 3.6.1. Oxidative Stress

One of the primary mechanisms by which mycotoxins exert toxicity is by inducing oxidative stress. Many mycotoxins stimulate the excessive production of reactive oxygen species (ROS) within cells, leading to an imbalance between oxidant generation and antioxidant defense systems [189]. Elevated ROS levels can damage cellular components such as lipids, proteins, and nucleic acids, ultimately impairing normal cellular function [190]. Lipid peroxidation of cellular membranes can compromise membrane integrity, while oxidative DNA damage may contribute to mutations and carcinogenesis [191]. The persistent oxidative stress induced by mycotoxins is therefore a key factor in their toxicity and carcinogenicity.

#### 3.6.2. Apoptosis Pathways

Mycotoxins can also trigger apoptosis, a programmed form of cell death that plays an essential role in maintaining tissue homeostasis [192]. Certain mycotoxins activate signaling pathways that lead to the activation of caspases, a family of proteolytic enzymes responsible for executing apoptosis. This activation can occur through both intrinsic (mitochondrial) and extrinsic (death receptor-mediated) pathways [193]. The induction of apoptosis in vital tissues such as the liver, kidneys, and immune cells contributes to organ damage and immunosuppression observed in mycotoxin exposure [194]. Prolonged activation of apoptotic pathways may also impair tissue regeneration and increase susceptibility to disease.

#### 3.6.3. Mitochondrial Dysfunction

Mitochondria are critical organelles responsible for energy production and the regulation of cellular metabolism [195]. Many mycotoxins interfere with mitochondrial function by disrupting electron transport chains, altering membrane potential, and promoting the release of pro-apoptotic factors such as cytochrome c. These disruptions reduce cellular energy production and increase reactive oxygen species generation, further amplifying oxidative stress [196]. Mitochondrial damage is therefore closely linked to both metabolic dysfunction and the activation of apoptotic pathways during mycotoxin toxicity [197,198].

#### 3.6.4. Inflammatory Signaling

**Figure 3:**
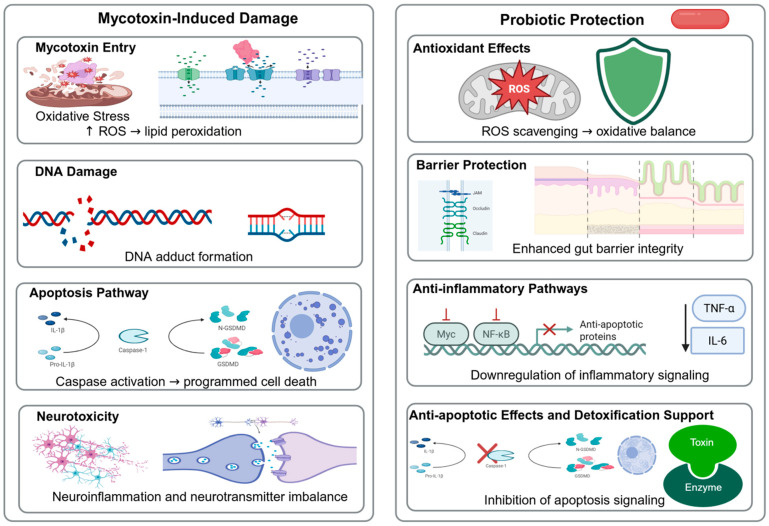
Cellular signaling pathways affected by mycotoxins and potential probiotic protection (Created with BioRender. Dietrich B. (2026)).

Another important consequence of mycotoxin exposure is the activation of inflammatory signaling pathways. Mycotoxins can stimulate the production of pro-inflammatory cytokines and activate transcription factors such as nuclear factor kappa B (NF-κB), which regulate immune and inflammatory responses [199]. Chronic activation of these pathways can lead to persistent inflammation, tissue injury, and impaired immune function. Inflammatory signaling also contributes to the progression of various diseases associated with mycotoxin exposure, including liver damage, gastrointestinal disorders, and immune dysregulation [200]. Understanding these molecular effects is essential for developing strategies to mitigate the health risks associated with mycotoxin contamination in food systems. Some studies have reported the protective effects of LAB-derived metabolites and probiotic preparations against mycotoxin-induced cellular damage in in vitro models, including Caco-2 intestinal epithelial cells, HepG2 hepatocyte models, and other intestinal epithelial systems. However, these findings are largely derived from controlled laboratory conditions and are often indirect in nature, focusing on biomarkers of oxidative stress, apoptosis, or inflammatory modulation rather than direct clinical endpoints [201,202,203]. In addition, relatively few studies have validated these protective effects in vivo, and the precise molecular mechanisms underlying the observed cellular protection remain incompletely understood [204]. Therefore, while LAB metabolites show promising protective potential against mycotoxin-induced cellular damage, further mechanistic and translational studies are required to establish causality and clinical relevance. The major cellular signaling pathways disrupted by mycotoxins, together with potential protective effects of probiotics, are presented in Figure 3.

### 3.7. Case Studies and Experimental Evidence

Scientific investigations over the past decades have provided substantial experimental evidence supporting the role of probiotic lactic acid bacteria (LAB) in controlling mycotoxin contamination and improving food safety. These studies encompass laboratory experiments, fermentation trials, and applied food system investigations that demonstrate the ability of LAB to detoxify mycotoxins, inhibit fungal growth, and enhance the preservation of food and beverages. The integration of microbiological, biochemical, and toxicological approaches in these studies has significantly expanded our understanding of the mechanisms through which LAB contribute to safer food systems.

#### 3.7.1. Aflatoxin Detoxification by LAB

Aflatoxins, particularly aflatoxin B_1_, are among the most toxic and carcinogenic mycotoxins found in food products such as cereals, nuts, and spices. Numerous experimental studies have demonstrated that specific LAB strains can reduce aflatoxin levels through adsorption, enzymatic degradation, or metabolic transformation [205]. For instance, strains of *Lactobacillus rhamnosus*, *Lactobacillus plantarum*, and *Lactobacillus casei* have shown strong binding affinity for aflatoxin B_1_, effectively reducing its bioavailability in contaminated food matrices [206]. Laboratory-based detoxification studies have revealed that LAB cell wall components, including peptidoglycan and polysaccharides, play a crucial role in aflatoxin adsorption [207,208,209]. In some cases, detoxification efficiencies ranging from 40% to over 80% have been reported depending on the bacterial strain, toxin concentration, and environmental conditions [210,211,212]. In addition to adsorption, certain LAB strains have been shown to enzymatically modify aflatoxin molecules into less toxic derivatives [213]. These findings highlight the promising role of LAB as biological agents for reducing aflatoxin exposure in food systems.

#### 3.7.2. Antifungal Metabolites in Fermented Foods

Fermented foods provide a natural environment for the production of antimicrobial and antifungal metabolites by LAB. Experimental studies investigating traditional fermented foods have revealed that LAB produce a variety of compounds that inhibit fungal growth and reduce the risk of mycotoxin formation. These metabolites include organic acids, bacteriocins, hydrogen peroxide, and volatile compounds such as diacetyl and acetoin [214]. Research on cereal-based fermentations and plant-derived beverages has shown that LAB can suppress the growth of toxigenic fungi, including *Aspergillus* spp., *Fusarium* spp., and *Penicillium* spp. The antifungal activity observed in these systems is often attributed to the combined effects of acidification, nutrient competition, and the production of antimicrobial metabolites [124]. These mechanisms contribute to the stability of fermented foods and limit fungal colonization during processing and storage. Consequently, fermented foods represent valuable natural systems for studying the antifungal potential of probiotic microorganisms.

#### 3.7.3. Probiotic Preservation of Beverages

The use of probiotic LAB in beverage preservation has also been widely explored as an alternative to chemical preservatives [215]. Experimental studies on both traditional and modern beverages have demonstrated that LAB fermentation can significantly improve microbial stability and extend shelf life. During fermentation, LAB produce organic acids and other antimicrobial compounds that inhibit spoilage microorganisms and pathogenic bacteria [216]. In traditional beverages such as cereal-based drinks and plant infusions, the introduction of selected probiotic strains has been shown to reduce microbial contamination and enhance product stability during storage [217]. Additionally, LAB-driven fermentation enhances sensory characteristics, including flavor development and aroma formation. These benefits make probiotic preservation an attractive strategy for maintaining beverage quality while ensuring food safety [218]. Overall, experimental evidence strongly supports the potential of LAB-based approaches as natural preservation strategies in food and beverage systems.

## 4. Innovations, Challenges, Limitations, and Future Perspectives

The application of probiotic LAB in food safety and mycotoxin control represents a rapidly evolving area of research that combines advances in microbiology, biotechnology, and toxicology. While significant progress has been made in understanding the antifungal and detoxification mechanisms of LAB, several scientific and technological challenges remain. Addressing these challenges will be essential for translating laboratory findings into large-scale industrial applications and ensuring the safety and reliability of LAB-based food preservation systems.

### 4.1. Innovations

Recent innovations in microbial biotechnology have expanded the potential applications of LAB in mycotoxin control and food preservation. One major advancement involves the development of microbial detoxification strategies that exploit the metabolic capabilities of probiotic microorganisms to degrade or bind harmful toxins [219,220]. These strategies include the identification of LAB strains with enhanced detoxification abilities, as well as the optimization of fermentation conditions to maximize toxin reduction in contaminated food matrices [221]. Another important innovation is the use of probiotic food preservation systems, which utilize beneficial microorganisms to extend shelf life and prevent microbial spoilage. By replacing or reducing chemical preservatives, these biological preservation strategies align with consumer demand for natural and clean-label food products [222]. The incorporation of LAB into fermentation processes not only improves microbial safety but also enhances nutritional and sensory qualities. Furthermore, recent research has emphasized the integration of toxicology and microbiology in the study of mycotoxin mitigation. This interdisciplinary approach allows researchers to investigate not only how microorganisms reduce toxin levels but also how detoxification processes influence cellular toxicity, metabolic pathways, and human health outcomes [223]. Such integrative research frameworks are essential for developing comprehensive strategies for mycotoxin risk management in food systems.

### 4.2. Challenges

Despite promising research outcomes, several challenges hinder the widespread application of LAB-based detoxification strategies in food systems. One of the most significant challenges is strain variability, as the antifungal and detoxification capabilities of LAB can differ considerably among species and even among strains within the same species. This variability complicates the selection of effective probiotic strains for specific food applications [66,224]. Another major challenge involves scaling fermentation processes from laboratory conditions to industrial production. Fermentation parameters such as temperature, pH, substrate composition, and microbial interactions must be carefully optimized to ensure consistent performance at larger scales. Maintaining the viability and metabolic activity of probiotic strains during large-scale processing and storage can also be difficult [225]. Additionally, verification of toxin degradation remains a critical challenge. In some cases, mycotoxin binding or transformation may produce intermediate compounds whose toxicity is not fully understood. Therefore, rigorous analytical and toxicological assessments are necessary to confirm that detoxification processes truly reduce toxicity rather than merely altering toxin structure [226].

Comprehensive toxicological validation is therefore essential to support claims of detoxification efficacy. This includes the use of LC–MS/MS for precise identification and quantification of parent mycotoxins and their transformation products, as well as in vitro cytotoxicity assays to evaluate cellular safety [227]. In addition, metabolomics-based approaches are increasingly important for characterizing global biochemical changes induced by LAB–mycotoxin interactions and for detecting unintended metabolic by-products that may not be captured by targeted analyses [228].

### 4.3. Limitations

Although the use of LAB in mycotoxin mitigation shows considerable promise, certain limitations must be acknowledged. One key limitation is the limited availability of clinical data demonstrating the health benefits of microbial detoxification in human populations. While numerous laboratory and animal studies have reported positive results, human-based studies evaluating the effectiveness of LAB in reducing mycotoxin exposure remain relatively scarce [229]. Another limitation relates to the variability in microbial efficacy observed across different food matrices and environmental conditions. Factors such as pH, temperature, water activity, and substrate composition can influence the performance of probiotic strains. As a result, a LAB strain that demonstrates strong detoxification activity in one food system may not exhibit the same effectiveness in another. This variability highlights the need for context-specific research and validation [230].

### 4.4. Regulatory Considerations

The application of probiotic microorganisms in food preservation and toxin mitigation must comply with strict regulatory frameworks designed to ensure consumer safety. Regulatory approval processes often require extensive evaluation of microbial strains, including their safety profile, genetic stability, and absence of pathogenic traits. Therefore, food safety approval represents a critical step in the commercialization of LAB-based preservation technologies [231].

Barriers to Regulatory Approval of LAB-Based Detoxification Products: Despite extensive experimental evidence supporting the antifungal and mycotoxin-detoxifying potential of lactic acid bacteria (LAB), the translation of these findings into commercially approved food applications remains limited. A major regulatory challenge is the lack of standardized protocols for evaluating microbial detoxification efficacy across diverse food matrices and processing conditions [62]. Considerable methodological variability exists among studies with respect to inoculum concentration, incubation parameters, toxin quantification techniques, and endpoints used to assess detoxification efficiency [232,233]. This lack of harmonization complicates inter-study comparability and limits the establishment of reproducible performance criteria required for regulatory assessment. In addition, the detoxification activity of LAB is highly strain-specific and mechanistically diverse. Closely related strains may differ substantially in their ability to adsorb, biotransform, or enzymatically degrade mycotoxins due to variations in cell wall composition, metabolic activity, and gene expression profiles. Consequently, regulatory approval cannot be generalized at the species level and instead requires strain-by-strain evaluation supported by robust phenotypic and genomic characterization [206]. This requirement significantly increases the complexity and cost of regulatory validation. Another critical limitation involves the insufficient toxicological characterization of mycotoxin transformation products generated during microbial detoxification. Although several studies have reported reductions in detectable toxin concentrations following LAB treatment, relatively few studies have confirmed that the resulting metabolites are safe under physiological conditions [234]. Regulatory authorities increasingly require integrated analytical and toxicological validation, including LC–MS/MS-based metabolite identification, cytotoxicity assays, genotoxicity assessment, and in vivo safety studies, to verify that biotransformation products do not retain mutagenic, carcinogenic, or immunotoxic properties. The lack of such confirmatory evidence remains a significant impediment to regulatory acceptance. Concerns also persist regarding the use of live microbial detoxifiers in food systems, particularly in relation to genomic stability, ecological persistence, and the potential horizontal transfer of antimicrobial resistance determinants [235]. As a result, genome-level safety assessment using whole-genome sequencing (WGS) has become increasingly important for detecting virulence-associated genes, antimicrobial resistance genes, plasmid-borne elements, and genomic instability that may compromise food safety. These genomic evaluations are also central to regulatory classification frameworks such as Qualified Presumption of Safety (QPS) in the European Food Safety Authority (EFSA) system and Generally Recognized as Safe (GRAS) status in the United States, both of which require robust evidence of microbial safety and genetic integrity [236]. Furthermore, most currently available evidence has been generated under controlled laboratory conditions, whereas industrial-scale validation in complex food systems remains limited. Variations in pH, water activity, food composition, storage conditions, and microbial interactions may significantly influence LAB functionality and detoxification efficiency during commercial processing [237]. Therefore, large-scale validation studies conducted under realistic manufacturing and storage conditions are essential to support regulatory approval and facilitate the industrial implementation of LAB-based mycotoxin mitigation technologies.

Another important aspect involves microbial strain certification, which ensures that the strains used in food production meet established safety and quality standards. Certification may involve documentation of strain identity, evaluation of antimicrobial resistance patterns, and verification of functional properties relevant to food safety [238,239]. Finally, the implementation of LAB-based detoxification strategies must align with international regulatory guidelines governing food additives, probiotics, and microbial processing aids. Organizations such as national food safety authorities and international regulatory bodies provide frameworks for evaluating the safety and efficacy of microbial interventions in food systems [240]. Adherence to these guidelines is essential for ensuring that probiotic-based preservation technologies can be safely and widely adopted within the global food industry.

## 5. Conclusions

This review underscores the emerging potential of probiotic lactic acid bacteria (LAB) isolated from indigenous fermented foods as multifunctional biological agents to control mycotoxin-producing fungi and mitigate associated health risks. Synthesizing evidence from microbial ecology, antifungal mechanisms, and detoxification pathways, this review demonstrates that LAB employ a range of strategies, including organic acid production, bacteriocin secretion, competitive exclusion, adsorption to cell wall components, and enzymatic transformation of mycotoxins, to inhibit fungal growth and reduce toxin levels across diverse food matrices. Beyond their role in food preservation, accumulating research indicates that LAB-derived metabolites can modulate cellular responses to mycotoxins, attenuating oxidative stress, inflammatory signaling, and apoptosis pathways in mammalian cells. These multifunctional properties position LAB as promising interventions that simultaneously improve food safety, nutritional quality, and consumer health. However, several important limitations must be acknowledged. The effects of LAB are highly strain-specific, with substantial variability in antifungal and detoxification performance even within closely related species. In addition, limited clinical and in vivo data currently restrict the ability to confirm physiological relevance in humans, as most evidence is derived from in vitro systems. Food matrix variability further complicates translation, since pH, water activity, nutrient composition, and processing conditions can significantly influence LAB activity and detoxification efficiency. Moreover, regulatory uncertainty regarding safety validation, genomic characterization, and approval pathways continues to hinder large-scale commercialization and standardization of LAB-based interventions. Future studies should focus on omics-based characterization of microbial detoxification pathways, validation of probiotic efficacy in real food systems, and translational investigations to assess physiological and cellular outcomes of mycotoxin mitigation. Moreover, research efforts that bridge microbiology, toxicology, and food technology will be critical for advancing regulatory acceptance and enabling scalable, safe, and sustainable LAB-based strategies to address persistent global challenges posed by fungal toxins in food systems.

## Figures and Tables

**Table 1:** Major Mycotoxins in Food Systems.

Fungal Species	Mycotoxin Produced	Food Sources	Health Effects	Example EU Maximum Levels by Food Category	Reference
*Aspergillus flavus*, *Aspergillus parasiticus*	Aflatoxins (AFB_1_, AFB_2_, AFG_1_, AFG_2_)	Peanuts, maize, tree nuts, spices	Hepatotoxicity, liver cancer, immunosuppression	2–12 µg/kg	[93]
*Fusarium verticillioides*, *Fusarium proliferatum*	Fumonisins (FB_1_, FB_2_)	Maize and maize products	Esophageal cancer, neural tube defects	400–4000 µg/kg	[94]
*Fusarium graminearum*, *Fusarium culmorum*	Deoxynivalenol (DON)	Wheat, barley, maize	Gastrointestinal disorders, immune modulation	750–1750 µg/kg	[95]
*Fusarium graminearum*, *Fusarium roseum*	Zearalenone	Maize, wheat, barley	Estrogenic effects, reproductive disorders	20–400 µg/kg	[96]
*Penicillium verrucosum*, *Aspergillus ochraceus*	Ochratoxin A	Cereals, coffee, dried fruits, wine	Nephrotoxicity, carcinogenic potential	3–10 µg/kg	[97]
*Penicillium expansum*	Patulin	Apples, apple juice, fruit products	Gastrointestinal toxicity, immune effects	50 µg/kg	[98]

**Table 2:** LAB Species from Fermented Foods with Antifungal Activity.

LAB Species	Food Source	Target Fungi	Antifungal Metabolites	Reported Efficacy	Reference
*Lactobacillus plantarum*	Ogi, garri	*Aspergillus flavus*, *Penicillium expansum*	Lactic acid, phenyllactic acid, bacteriocins	Significant inhibition of fungal growth and reduced aflatoxin production	[123]
*Lactobacillus fermentum*	Ogi, fufu	*Fusarium verticillioides*, *Aspergillus parasiticus*	Organic acids, hydrogen peroxide	Suppression of fungal growth and toxin production	[124]
*Lactococcus lactis*	Fermented cereals, zobo	*Penicillium* sp., *Aspergillus* sp.	Nisin, organic acids	Inhibition of fungal proliferation in fermented beverages	[125]
*Weissella confusa*	Ogi, fermented cassava	*Fusarium* sp., *Aspergillus flavus*	Organic acids, antimicrobial peptides	Moderate antifungal activity and growth suppression	[126]
*Pediococcus acidilactici*	Garri, fermented grains	*Penicillium* sp., *Fusarium* sp.	Pediocin, lactic acid	Reduced fungal colonization in fermented substrates	[127]
*Leuconostoc mesenteroides*	Fermented cassava, ogi	*Aspergillus* sp., *Penicillium* sp.	Diacetyl, organic acids	Inhibition of fungal growth and spoilage microorganisms	[128]

**Table 3:** Microbial Detoxification Mechanisms.

Mycotoxin	LAB Species	Detoxification Mechanism	Enzyme Involved	Reduction (%)	Reference
Aflatoxin B1	*Lactobacillus rhamnosus*	Cell wall adsorption	Peptidoglycan-associated binding	40–80%	[159]
Aflatoxin B1	*Lactobacillus plantarum*	Enzymatic degradation	Oxidoreductase	30–70%	[160]
Ochratoxin A	*Lactobacillus acidophilus*	Enzymatic hydrolysis	Esterase	25–60%	[161]
Zearalenone	*Lactobacillus casei*	Lactone ring cleavage	Lactonase	30–65%	[162]
Patulin	*Lactobacillus brevis*	Metabolite transformation	Oxidoreductase	20–55%	[163]
Fumonisin B1	*Lactobacillus fermentum*	Enzymatic hydrolysis	Esterase	20–50%	[164]

**Table 4:** Applications of LAB for Mycotoxin Control in Food.

Food Matrix	LAB Strain	Application Method	Target Toxin	Outcome	Reference
Peanut and Sun flower cake	*Lactobacillus plantarum*	Controlled fermentation	Aflatoxin B1	Reduced fungal growth and improved shelf life	[181]
Maize based fermented food	*Lactobacillus rhamnosus*	Starter culture inoculation	Ochratoxin A	Partial detoxification and microbial stabilization	[182]
Soy-based dairy analogue	*Lactobacillus acidophilus*	Probiotic fermentation	Aflatoxin B1	Reduced toxin bioavailability	[183]
Almond-based dairy analogue	*Lactobacillus casei*	Fermentation starter	Zearalenone	Reduced fungal contamination	[184]
Fermented maize (cereal fermentation)	*Lactobacillus fermentum*	Natural fermentation	Fumonisin B1	Lower toxin levels and improved safety	[185]
Sorghum fermentation	*Lactobacillus brevis*	Starter culture fermentation	Aflatoxin B1	Suppression of toxigenic fungi	[186]

## Data Availability

No new data were created or analyzed in this study. Data sharing is not applicable to this article.

## Abbreviations

LAB	Lactic Acid Bacteria
OTA	Ochratoxin A
AFB1	Aflatoxin B1
ROS	Reactive Oxygen Species
SCFA	Short-Chain Fatty Acids
HPLC	High-Performance Liquid Chromatography
LC-MS	Liquid Chromatography Mass Spectrometry
GRAS	Generally Recognized As Safe

## References

[1] Yilmaz N., Verheecke-Vaessen C., Ezekiel C.N. (2025). Mycotoxins: An ongoing challenge to food safety and security. PLoS Pathog..

[2] Long N., Peng H., Wang Y., Zhou C., Wang Z. (2025). Effects of mycotoxins in foods on human health and strategies for prevention and control: A review. J. Future Foods.

[3] Shekhar R., Raghavendra V.B., Rachitha P. (2025). A comprehensive review of mycotoxins, their toxicity, and innovative detoxification methods. Toxicol. Rep..

[4] Syamilah N., Nurul Afifah S., Effarizah M.E., Norlia M. (2022). Mycotoxins and mycotoxigenic fungi in spices and mixed spices: A review. Food Res..

[5] Bullerman L.B., Bianchini A. (2007). Stability of mycotoxins during food processing. Int. J. Food Microbiol..

[6] Molina-Hernandez J.B., Grande-Tovar C.D., Neri L., Delgado-Ospina J., Rinaldi M., Cordero-Bueso G.A., Chaves-López C. (2025). Enhancing postharvest food safety: The essential role of non-thermal technologies in combating fungal contamination and mycotoxins. Front. Microbiol..

[7] Karlovsky P., Suman M., Berthiller F., De Meester J., Eisenbrand G., Perrin I., Oswald I.P., Speijers G., Chiodini A., Recker T. (2016). Impact of food processing and detoxification treatments on mycotoxin contamination. Mycotoxin Res..

[8] Mafe A.N., Nkene I.H., Ali A.B.M., Edo G.I., Akpoghelie P.O., Yousif E., Isoje E.F., Igbuku U.A., Ismael S.A., Essaghah A.E.A. (2025). Smart Probiotic Solutions for Mycotoxin Mitigation: Innovations in Food Safety and Sustainable Agriculture. Probiotics Antimicrob. Proteins.

[9] Awuchi C.G., Ondari E.N., Ogbonna C.U., Upadhyay A.K., Baran K., Okpala C.O.R., Korzeniowska M., Guiné R.P.F. (2021). Mycotoxins Affecting Animals, Foods, Humans, and Plants: Types, Occurrence, Toxicities, Action Mechanisms, Prevention, and Detoxification Strategies—A Revisit. Foods.

[10] Niaz W., Iqbal S.Z., Ahmad K., Majid A., Haider W., Li X. (2025). Mycotoxins: A comprehensive review of its global trends in major cereals, advancements in chromatographic detections and future prospectives. Food Chem. X.

[11] Groopman J.D., Smith J.W., Rivera-Andrade A., Alvarez C.S., Kroker-Lobos M.F., Egner P.A., Gharzouzi E., Dean M., McGlynn K.A., Ramírez-Zea M. (2021). Aflatoxin and the aetiology of liver cancer and its implications for Guatemala. World Mycotoxin J..

[12] Chen J.-G., Kensler T.W., Sun G.-J., Zhu J., Lu J.-H., Pan D., Zhang Y.-H., Groopman J.D. (2026). Aflatoxin and Liver Cancer in China: The Evolving Research Landscape. Toxins.

[13] Akinmoladun O.F., Fon F.N., Nji Q., Adeniji O.O., Tangni E.K., Njobeh P.B. (2025). Multiple Mycotoxin Contamination in Livestock Feed: Implications for Animal Health, Productivity, and Food Safety. Toxins.

[14] Shanmugasundaram R. (2025). Current approaches to the ongoing challenges of mycotoxins in poultry diets: Understanding and combating mycotoxins for sustainable poultry production. J. Appl. Poult. Res..

[15] Goda A.A., Shi J., Xu J., Liu X., Zhou Y., Xiao L., Abdel-Galil M., Salem S.H., Ayad E.G., Deabes M. (2025). Global health and economic impacts of mycotoxins: A comprehensive review. Environ. Sci. Eur..

[16] Stoev S.D. (2024). Food security, underestimated hazard of joint mycotoxin exposure and management of the risk of mycotoxin contamination. Food Control.

[17] Leyva Salas M., Mounier J., Valence F., Coton M., Thierry A., Coton E. (2017). Antifungal Microbial Agents for Food Biopreservation—A Review. Microorganisms.

[18] Sánchez-Torres P. (2025). Emerging alternatives to control fungal contamination. Curr. Opin. Food Sci..

[19] Mesías F.J., Martín A., Hernández A. (2021). Consumers’ growing appetite for natural foods: Perceptions towards the use of natural preservatives in fresh fruit. Food Res. Int..

[20] Guzik P., Szymkowiak A., Kulawik P., Zając M. (2022). Consumer Attitudes towards Food Preservation Methods. Foods.

[21] Lisboa H.M., Pasquali M.B., dos Anjos A.I., Sarinho A.M., de Melo E.D., Andrade R., Batista L., Lima J., Diniz Y., Barros A. (2024). Innovative and Sustainable Food Preservation Techniques: Enhancing Food Quality, Safety, and Environmental Sustainability. Sustainability.

[22] Bento de Carvalho T., Silva B.N., Tomé E., Teixeira P. (2024). Preventing Fungal Spoilage from Raw Materials to Final Product: Innovative Preservation Techniques for Fruit Fillings. Foods.

[23] Garnier L., Valence F., Mounier J. (2017). Diversity and Control of Spoilage Fungi in Dairy Products: An Update. Microorganisms.

[24] Teshome E., Forsido S.F., Rupasinghe H.P.V., Olika Keyata E. (2022). Potentials of Natural Preservatives to Enhance Food Safety and Shelf Life: A Review. Sci. World J..

[25] Mohamed A.B., Gathman R.J., Chavez R.A., Wagacha M.J., Mutegi C.K., Muthomi J.W., Stasiewicz M.J. (2023). Multispectral Sorting Based on Visibly High-Risk Kernels Sourced from Another Country Reduces Fumonisin and Toxigenic Fusarium on Maize Kernels. J. Food Prot..

[26] Conte G., Fontanelli M., Galli F., Cotrozzi L., Pagni L., Pellegrini E. (2020). Mycotoxins in Feed and Food and the Role of Ozone in Their Detoxification and Degradation: An Update. Toxins.

[27] Yun S.C., Jeong H., Lee J.-S., Kim J.-H., Kim I.-C., Maszczyk P., Yang Z., Hagiwara A., Lee J.-S. (2026). A review of ammonia toxicity on aquatic organisms: Species-specific responses, microbial shifts, and environmental interactions. Comp. Biochem. Physiol. Part C Toxicol. Pharmacol..

[28] Sun H., He Z., Xiong D., Long M. (2023). Mechanisms by which microbial enzymes degrade four mycotoxins and application in animal production: A review. Anim. Nutr..

[29] Syraji Y., Jeyaramraja P.R., Mada T., Gobikanila K. (2025). Comprehensive review of aflatoxin contamination, its occurrence, effects, management, and future perspectives. Discov. Food.

[30] Ji J., Jiang X., Song P., Yang Q., Sun M., Dong Z., Lu Y., Dou S., Dong L. (2025). Multi-Omics Insights into Microbial Interactions and Fermented Food Quality. Microorganisms.

[31] Tamang J.P., Watanabe K., Holzapfel W.H. (2016). Review: Diversity of Microorganisms in Global Fermented Foods and Beverages. Front. Microbiol..

[32] Obayomi O.V., Edo G.I. (2025). Exploring the health benefits, mechanisms of action, and emerging safety concerns of fermented foods with emphasis on African foods. Food Wellness.

[33] Oguntoyinbo F.A., Fusco V., Cho G.-S., Kabisch J., Neve H., Bockelmann W., Huch M., Frommherz L., Trierweiler B., Becker B. (2016). Produce from Africa’s Gardens: Potential for Leafy Vegetable and Fruit Fermentations. Front. Microbiol..

[34] Abdul Hakim B.N., Xuan N.J., Oslan S.N.H. (2023). A Comprehensive Review of Bioactive Compounds from Lactic Acid Bacteria: Potential Functions as Functional Food in Dietetics and the Food Industry. Foods.

[35] Fitsum S., Gebreyohannes G., Sbhatu D.B. (2025). Bioactive compounds in fermented foods: Health benefits, safety, and future perspectives. Appl. Food Res..

[36] Alves V., Zamith-Miranda D., Frases S., Nosanchuk J.D. (2025). Fungal Metabolomics: A Comprehensive Approach to Understanding Pathogenesis in Humans and Identifying Potential Therapeutics. J. Fungi.

[37] Saez J.M., Raimondo E.E., Costa-Gutierrez S.B., Aparicio J.D., Mosca Angelucci D., Donati E., Polti M.A., Tomei M.C., Benimeli C.S. (2025). Enhancing environmental decontamination and sustainable production through synergistic and complementary interactions of actinobacteria and fungi. Heliyon.

[38] Castellone V., Bancalari E., Rubert J., Gatti M., Neviani E., Bottari B. (2021). Eating Fermented: Health Benefits of LAB-Fermented Foods. Foods.

[39] Ahansaz N., Tarrah A., Pakroo S., Corich V., Giacomini A. (2023). Lactic Acid Bacteria in Dairy Foods: Prime Sources of Antimicrobial Compounds. Fermentation.

[40] Edo G.I., Mafe A.N., Ali A.B.M., Akpoghelie P.O., Yousif E., Lwanegbe L., Igbuku U.A., Owheruo J.O., Essaghah A.E.A., Ahmed D.S. (2026). Probiotics as eco-friendlybio-preservatives: In vivo mechanisms of mycotoxin inhibition and emerging applications for food safety and human health. Mycotoxin Res..

[41] Amalaradjou M.A.R., Bhunia A.K. (2012). Chapter Five—Modern Approaches in Probiotics Research to Control Foodborne Pathogens. Advances in Food and Nutrition Research.

[42] Ramírez A., Bermúdez-Luque A., Román-Camacho J.J., Martín-García F.J., Moreno-García J., Güngörmüşler M., Ruiz-Castilla F.J. (2025). Exploring the future of probiotics with innovations in delivery systems and market insights. Food Biosci..

[43] Teneva D., Denev P. (2023). Biologically Active Compounds from Probiotic Microorganisms and Plant Extracts Used as Biopreservatives. Microorganisms.

[44] Vinayamohan P., Joseph D., Viju L.S., Baskaran S.A., Venkitanarayanan K. (2024). Efficacy of Probiotics in Reducing Pathogenic Potential of Infectious Agents. Fermentation.

[45] Divyashree S., Shruthi B., Vanitha P.R., Sreenivasa M.Y. (2023). Probiotics and their postbiotics for the control of opportunistic fungal pathogens: A review. Biotechnol. Rep..

[46] Zhang Y., Li L., Pang X., Zhang S., Liu Y., Wang Y., Xie N., Li X. (2026). Lactic Acid Bacteria as the Green and Safe Food Preservatives: Their Mechanisms, Applications and Prospects. Foods.

[47] Petrova P., Arsov A., Tsvetanova F., Parvanova-Mancheva T., Vasileva E., Tsigoriyna L., Petrov K. (2022). The Complex Role of Lactic Acid Bacteria in Food Detoxification. Nutrients.

[48] Choi D., Fan X., Yu J.-H. (2025). Comprehensive Review of Dietary Probiotics in Reducing Aflatoxin B1 Toxicity. Toxins.

[49] Mafe A.N., Büsselberg D. (2026). Microbiome–mycotoxin interactions and probiotic strategies: Implications for gut health and cancer. Front. Nutr..

[50] Nahle S., El Khoury A., Savvaidis I., Chokr A., Louka N., Atoui A. (2022). Detoxification approaches of mycotoxins: By microorganisms, biofilms and enzymes. Int. J. Food Contam..

[51] Awuchi C.G., Ondari E.N., Nwozo S., Odongo G.A., Eseoghene I.J., Twinomuhwezi H., Ogbonna C.U., Upadhyay A.K., Adeleye A.O., Okpala C.O.R. (2022). Mycotoxins’ Toxicological Mechanisms Involving Humans, Livestock and Their Associated Health Concerns: A Review. Toxins.

[52] Mafe A.N., Büsselberg D. (2024). Mycotoxins in Food: Cancer Risks and Strategies for Control. Foods.

[53] Janik E., Niemcewicz M., Ceremuga M., Stela M., Saluk-Bijak J., Siadkowski A., Bijak M. (2020). Molecular Aspects of Mycotoxins—A Serious Problem for Human Health. Int. J. Mol. Sci..

[54] Yao C., Ye M., Wang C., Zou L., Zhang X., Chai X., Yu H., Zhang C., Wang Y. (2025). Mycotoxin Contamination: Occurrence, Biotransformation, Pathogenic Mechanisms, and Strategies for Nutritional Intervention. Molecules.

[55] Jobe M.C., Mthiyane D.M.N., Dludla P.V., Mazibuko-Mbeje S.E., Onwudiwe D.C., Mwanza M. (2023). Pathological Role of Oxidative Stress in Aflatoxin-Induced Toxicity in Different Experimental Models and Protective Effect of Phytochemicals: A Review. Molecules.

[56] Tao Y., Xie S., Xu F., Liu A., Wang Y., Chen D., Pan Y., Huang L., Peng D., Wang X. (2018). Ochratoxin A: Toxicity, oxidative stress and metabolism. Food Chem. Toxicol..

[57] Abia W.A., Foupouapouognigni Y., Nfombouot H.P.N., Ngoungoure L.V.N., Ntungwe E.N., Salah-Abbès J.B., Tchana A.N. (2025). A scoping review on mycotoxin-induced neurotoxicity. Discov. Toxicol..

[58] Yao J., Ouyang B., Xu W., Xie Y., Mu W. (2026). An overview of the physical, chemical and biological strategies for the removal of emerging mycotoxins: Recent advances and future perspectives. Food Control.

[59] Wang C., Youle R.J. (2009). The Role of Mitochondria in Apoptosis. Annu. Rev. Genet..

[60] Martinou J.-C., Youle R.J. (2011). Mitochondria in Apoptosis: Bcl-2 Family Members and Mitochondrial Dynamics. Dev. Cell.

[61] Islam M.M., Mahbub N.U., Islam M.A. (2024). Gut Microorganism-Mediated Neutralization of Mycotoxins: A Promising Approach to Combat Fungal Toxicity. Adv. Gut Microbiome Res..

[62] Aasa A., Govender S., Malgas S., Thantsha M. (2026). Microbial and enzymatic biodegradation of aflatoxins and ochratoxins: Mechanisms, applications, and emerging innovations. Arch. Microbiol..

[63] Hayek S.A., Ibrahim S.A. (2013). Current Limitations and Challenges with Lactic Acid Bacteria: A Review. Food Nutr. Sci..

[64] Van Rossum T., Ferretti P., Maistrenko O.M., Bork P. (2020). Diversity within species: Interpreting strains in microbiomes. Nat. Rev. Microbiol..

[65] Jauneikaite E., Baker K.S., Nunn J.G., Midega J.T., Hsu L.Y., Singh S.R., Halpin A.L., Hopkins K.L., Price J.R., Srikantiah P. (2023). Genomics for antimicrobial resistance surveillance to support infection prevention and control in health-care facilities. Lancet Microbe.

[66] Mateo F., Mateo E.M., Tarazona A., García-Esparza M.Á., Soria J.M., Jiménez M. (2025). New Strategies and Artificial Intelligence Methods for the Mitigation of Toxigenic Fungi and Mycotoxins in Foods. Toxins.

[67] Sangmanee P., Hongpattarakere T. (2014). Inhibitory of multiple antifungal components produced by *Lactobacillus plantarum* K35 on growth, aflatoxin production and ultrastructure alterations of *Aspergillus flavus* and *Aspergillus parasiticus*. Food Control.

[68] Loi M., Fanelli F., Liuzzi V., Logrieco A., Mulè G. (2017). Mycotoxin Biotransformation by Native and Commercial Enzymes: Present and Future Perspectives. Toxins.

[69] Ghosh S., Bornman C., Meskini M., Joghataei M. (2024). Microbial Diversity in African Foods and Beverages: A Systematic Assessment. Curr. Microbiol..

[70] Monteiro C.S., Pinto E., López-Ruiz R., Marín-Sáez J., Frenich A.G., Faria M.A., Cunha S.C. (2025). Integrated Assessment of Fungi Contamination and Mycotoxins Levels Across the Rice Processing Chain. Toxins.

[71] Kushwaha S., Soni H., Tandon S., Singh G., Gandhi Y., Kumar V., Jagtap C., Narasimhaji C.V., Mathapati S., Srikanth N. (2025). Fungal toxin (mycotoxin): Introduction, sources and types, production, detection, and applications. Food Nutr..

[72] Nji Q.N., Babalola O.O., Mwanza M. (2023). Soil Aspergillus Species, Pathogenicity and Control Perspectives. J. Fungi.

[73] Manizan A.L., Carvajal-Campos A., Piro-Metayer I., Akaki D.K., Koffi-Nevry R., Montet D., Oswald I.P., Lorber S., Puel O., Brabet C. (2026). Biodiversity of Aspergillus section Flavi species isolated along the peanut paste production chain in Côte d’Ivoire. Int. J. Food Microbiol..

[74] Shabeer S., Asad S., Jamal A., Ali A. (2022). Aflatoxin Contamination, Its Impact and Management Strategies: An Updated Review. Toxins.

[75] Obedi F.A., Mtei R.P., Kilulya K.F. (2025). Mitigating Aflatoxin Contamination in Groundnuts Using Plant-Derived Essential Oils: Implications for Food Security. J. Food Qual..

[76] Mohapatra D., Kumar S., Kotwaliwale N., Singh K.K. (2017). Critical factors responsible for fungi growth in stored food grains and non-Chemical approaches for their control. Ind. Crops Prod..

[77] Jedua H., Mafe A.N., Edo G.I., Ali A.B.M., Iwanegbe I., Akpoghelie P.O., Owheruo J.O., Onukwubiri M., Yousif E., Igbuku U.A. (2026). Microbial contamination pathways: Assessing the impact of building-damaging microorganisms on food safety and structural integrity in humid home environments. J. Stored Prod. Res..

[78] Qu Z., Ren X., Du Z., Hou J., Li Y., Yao Y., An Y. (2024). Fusarium mycotoxins: The major food contaminants. mLife.

[79] Ekwomadu T.I., Akinola S.A., Mwanza M. (2021). Fusarium Mycotoxins, Their Metabolites (Free, Emerging, and Masked), Food Safety Concerns, and Health Impacts. Int. J. Environ. Res. Public Health.

[80] El-Sayed R.A., Jebur A.B., Kang W., El-Demerdash F.M. (2022). An overview on the major mycotoxins in food products: Characteristics, toxicity, and analysis. J. Future Foods.

[81] Di Salvo E., Bartolomeo G., Vadalà R., Costa R., Cicero N. (2025). Mycotoxins in Ready-to-Eat Foods: Regulatory Challenges and Modern Detection Methods. Toxics.

[82] Perrone G., Susca A. (2017). *Penicillium* Species and Their Associated Mycotoxins. Mycotoxigenic Fungi. Methods in Molecular Biology.

[83] Mannaa M., Kim K.D. (2017). Influence of Temperature and Water Activity on Deleterious Fungi and Mycotoxin Production during Grain Storage. Mycobiology.

[84] Kovač Tomas M., Jurčević Šangut I. (2025). New Insights into Mycotoxin Contamination, Detection, and Mitigation in Food and Feed Systems. Toxins.

[85] Li Y., He Z., Zhao Y., Lv H., Han G. (2026). Temperature-Dependent Fungal Diversity, Storage Quality, and Processing Quality of High-Moisture Wheat During Post-Harvest Storage. Foods.

[86] Snyder A.B., Worobo R.W. (2018). Fungal Spoilage in Food Processing. J. Food Prot..

[87] Zheng Y., Wu W., Sun C., Liu H., Dou J. (2024). Occurrence and Fate Analysis of Mycotoxins in Maize During the Post-Harvest Period. Toxins.

[88] Zaman W., Amin A., Khalil A.A.K., Akhtar M.S., Ali S. (2025). Plant–Microbe Interactions for Improving Postharvest Shelf Life and Quality of Fresh Produce Through Protective Mechanisms. Horticulturae.

[89] Bradford K.J., Dahal P., Van Asbrouck J., Kunusoth K., Bello P., Thompson J., Wu F. (2018). The dry chain: Reducing postharvest losses and improving food safety in humid climates. Trends Food Sci. Technol..

[90] Deng X., Li H., Wu A., He J., Mao X., Dai Z., Tian G., Cai J., Tang J., Luo Y. (2025). Composition, Influencing Factors, and Effects on Host Nutrient Metabolism of Fungi in Gastrointestinal Tract of Monogastric Animals. Animals.

[91] Zhang C., Qu Z., Hou J., Yao Y. (2024). Contamination and Control of Mycotoxins in Grain and Oil Crops. Microorganisms.

[92] Pouris J., Kolyva F., Bratakou S., Vogiatzi C.A., Chaniotis D., Beloukas A. (2024). The Role of Fungi in Food Production and Processing. Appl. Sci..

[93] Sogbossi Gbétokpanou C., Jonard C., Mehinto O.A., Gofflot S., Adjéniya M.J.M., Iko Afe O.H., Anihouvi D.G., Boutaleb S., Bragard C., Azokpota P. (2025). Peanut and Peanut-Based Foods Contamination by Toxigenic Fungi and Mycotoxins: Potential Risks for Beninese Consumers. Toxins.

[94] Rosa Junior O., Dalcin M., Nascimento V., Haesbaert F., Ferreira T., Fidelis R., Sarmento R., Aguiar R., Oliveira E., Santos G. (2019). Fumonisin Production by Fusarium verticillioides in Maize Genotypes Cultivated in Different Environments. Toxins.

[95] Bobadilla-Meléndez M., Zamora-Díaz M.R., Hernández-Anguiano A.M. (2025). Fusarium graminearum quimiotipo Don en grano de cebada maltera cultivada en México. Rev. Mex. Cienc. Agríc..

[96] Jung B., Kim H.E., Lee J., Li T. (2025). Effects of β-estradiol on the Phytopathogenic Fungus Fusarium graminearum. Plant Pathol. J..

[97] Kosicki R., Buharowska-Donten J., Twarużek M. (2021). Ochratoxin A levels in serum of Polish dialysis patients with chronic renal failure. Toxicon.

[98] Elhariry H., Bahobial A.A., Gherbawy Y. (2011). Genotypic identification of *Penicillium expansum* and the role of processing on patulin presence in juice. Food Chem. Toxicol..

[99] Park I., Mannaa M. (2025). Fermented Foods as Functional Systems: Microbial Communities and Metabolites Influencing Gut Health and Systemic Outcomes. Foods.

[100] Obafemi Y.D., Oranusi S.U., Ajanaku K.O., Akinduti P.A., Leech J., Cotter P.D. (2022). African fermented foods: Overview, emerging benefits, and novel approaches to microbiome profiling. npj Sci. Food.

[101] Voidarou C., Antoniadou Μ., Rozos G., Tzora A., Skoufos I., Varzakas T., Lagiou A., Bezirtzoglou E. (2020). Fermentative Foods: Microbiology, Biochemistry, Potential Human Health Benefits and Public Health Issues. Foods.

[102] Banwo K., Oyeyipo A., Mishra L., Sarkar D., Shetty K. (2022). Improving phenolic bioactive-linked functional qualities of traditional cereal-based fermented food (Ogi) of Nigeria using compatible food synergies with underutilized edible plants. NFS J..

[103] Sharma R., Garg P., Kumar P., Bhatia S.K., Kulshrestha S. (2020). Microbial Fermentation and Its Role in Quality Improvement of Fermented Foods. Fermentation.

[104] Wafula E.N., Muhonja C.N., Kuja J.O., Owaga E.E., Makonde H.M., Mathara J.M., Kimani V.W. (2022). Lactic Acid Bacteria from African Fermented Cereal-Based Products: Potential Biological Control Agents for Mycotoxins in Kenya. J. Toxicol..

[105] Adeoye B.K., Oyewole O.B., Idowu M.A., Obadina A.O., Ani I.F., Ngozi E.O. (2018). Effect of spices on the microbiological quality of *Hibiscus sabdariffa* (zobo) drink and molecular characterization of the associated spoilage organisms. Afr. J. Biotechnol..

[106] Omotayo J.O., Ajibade O.A., Oyawoye O.M. (2024). Insight into the Beneficial Use of Iru An African Condiment from Parkia Biglobosa. Int. J. Res. Innov. Appl. Sci..

[107] Azokpota P., Hounhouigan D.J., Nago M.C. (2006). Microbiological and chemical changes during the fermentation of African locust bean (*Parkia biglobosa*) to produce afitin, iru and sonru, three traditional condiments produced in Benin. Int. J. Food Microbiol..

[108] Bayode A.A., Okhonlaye O.A. (2020). Microorganisms Associated with the Fermentation of Gari Fortified with Sprouted Mung Beans Flour. South Asian J. Res. Microbiol..

[109] Halake N.H., Chinthapalli B. (2020). Fermentation of Traditional African Cassava Based Foods: Microorganisms Role in Nutritional and Safety Value. J. Exp. Agric. Int..

[110] Guan Y., Lv H., Wu G., Chen J., Wang M., Zhang M., Pang H., Duan Y., Wang L., Tan Z. (2023). Effects of Lactic Acid Bacteria Reducing the Content of Harmful Fungi and Mycotoxins on the Quality of Mixed Fermented Feed. Toxins.

[111] Achi O.K., Akomas  N.S. (2006). Comparative Assessment of Fermentation Techniques in the Processing of Fufu, a Traditional Fermented Cassava Product. Pak. J. Nutr..

[112] Bamidele O.P. (2025). Effects of Natural Fermentation Time on Chemical Composition, Antioxidant Activities, and Phenolic Profile of Cassava Root Flour. Appl. Sci..

[113] Sawant S.S., Park H.-Y., Sim E.-Y., Kim H.-S., Choi H.-S. (2025). Microbial Fermentation in Food: Impact on Functional Properties and Nutritional Enhancement—A Review of Recent Developments. Fermentation.

[114] Rusu A.V., Trif M., Rocha J.M. (2023). Microbial Secondary Metabolites via Fermentation Approaches for Dietary Supplementation Formulations. Molecules.

[115] Abedi E., Hashemi S.M.B. (2020). Lactic acid production—Producing microorganisms and substrates sources-state of art. Heliyon.

[116] Parada Fabián J.C., Álvarez Contreras A.K., Natividad Bonifacio I., Hernández Robles M.F., Vázquez Quiñones C.R., Quiñones Ramírez E.I., Vázquez Salinas C. (2025). Toward safer and sustainable food preservation: A comprehensive review of bacteriocins in the food industry. Biosci. Rep..

[117] Amenu D., Nugusa A., Tafesse T. (2025). Preservative Effectiveness of Lactic Acid Bacteria on Fruits and Vegetables. Int. J. Cell Biol..

[118] De Simone N., López L., Ciudad C.S., Scauro A., Russo P., Rodríguez J., Spano G., Martínez B. (2024). Antifungal activity of *Lactiplantibacillus plantarum* isolated from fruit and vegetables and detection of novel antifungal VOCs from fungal-LAB co-cultures. Food Biosci..

[119] Mayirnao H.-S., Sharma K., Jangir P., Kaur S., Kapoor R. (2025). Mushroom-derived nutraceuticals in the 21st century: An appraisal and future perspectives. J. Future Foods.

[120] Hibbing M.E., Fuqua C., Parsek M.R., Peterson S.B. (2010). Bacterial competition: Surviving and thriving in the microbial jungle. Nat. Rev. Microbiol..

[121] Wang D., Zeng N., Li C., Li Z., Zhang N., Li B. (2024). Fungal biofilm formation and its regulatory mechanism. Heliyon.

[122] Raman J., Kim J.-S., Choi K.R., Eun H., Yang D., Ko Y.-J., Kim S.-J. (2022). Application of Lactic Acid Bacteria (LAB) in Sustainable Agriculture: Advantages and Limitations. Int. J. Mol. Sci..

[123] Guimarães A., Santiago A., Teixeira J.A., Venâncio A., Abrunhosa L. (2018). Anti-aflatoxigenic effect of organic acids produced by *Lactobacillus plantarum*. Int. J. Food Microbiol..

[124] Wu-Wu J.W.F., Barboza N., Villalta-Romero F., Viñas M. (2025). Antagonistic Potential of Agro-Industrial Byproduct–Derived Lactic Acid Bacteria Against Mycotoxigenic *Aspergillus flavus* and *Fusarium verticillioides*. Int. J. Microbiol..

[125] Zakari D., Amoka A., Idris E., Anoze A., Moyosore A., Boniface M., Bashir A., Danjuma S., Omuya A. (2025). Characterization of lactic acid bacteria from fermented cereal-based foods in Anyigba, Nigeria, for potential probiotic and bio-preservation applications. Agric. Food Bioact. Compd..

[126] Krishnan S.V., Anaswara P.A., Nampoothiri K.M., Kovács S., Adácsi C., Miklós I., Király S., Pócsi I., Pusztahelyi T. (2025). Unveiling the Perspective on *Weissella confusa* as a Promising Biocontrol Agent Against Fusaria. Microorganisms.

[127] Pongking T., Chen X., Tunbenjasiri K., Zhang L., Kraiklang R., Pinlaor S., Sangka A., Lulitanond A., Blair D., Pinlaor P. (2025). Impact of Fermentation on Bacterial and Fungal Microbiome Interactions in Pla-Ra, a Traditional Thai Food. Int. J. Food Sci..

[128] Bamigbade G.B. (2019). Isolation and Screening of Antifungal Producing Lactic Acid Bacteria From Pro-Vitamin A Cassava. J. Nat. Sci. Res..

[129] Liu A., Xu R., Zhang S., Wang Y., Hu B., Ao X., Li Q., Li J., Hu K., Yang Y. (2022). Antifungal Mechanisms and Application of Lactic Acid Bacteria in Bakery Products: A Review. Front. Microbiol..

[130] Kim T.H., Kwon C.W. (2026). Effect of anti-mold organic compounds on shelf-life extension and quality of yeast-fermented bread. LWT.

[131] Qiu J., Li Y., Zhang X., Wang L., Wang J., Jiang X., Li M., Chen X., Elena S., Chen L. (2026). Synergistic antibacterial activity and mechanism of ε-polylysine hydrochloride and sodium diacetate against *Serratia liquefaciens*. LWT.

[132] Dopazo V., Musto L., de Melo Nazareth T., Lafuente C., Meca G., Luz C. (2024). Revalorization of rice bran as a potential ingredient for reducing fungal contamination in bread by lactic acid bacterial fermentation. Food Biosci..

[133] Marín A., Plotto A., Atarés L., Chiralt A. (2019). Lactic Acid Bacteria Incorporated into Edible Coatings to Control Fungal Growth and Maintain Postharvest Quality of Grapes. HortScience.

[134] Nugrahani A.W., Hertiani T., Haniastuti T., Zai K. (2025). Anacardic acid as a promising natural antimicrobial agent: Mechanisms of action, biofilm inhibition, and advances in nano-encapsulation for enhanced therapeutic efficacy. Fitoterapia.

[135] Choi G.H., Fugaban J.I.I., Dioso C.M., Bucheli J.E.V., Holzapfel W.H., Todorov S.D. (2025). Antimicrobial Peptides (Bacteriocins) Produced by *Lactococcus lactis* and *Pediococcus pentosaceus* Strains with Activity Against Clinical and Food-Borne Pathogens. Probiotics Antimicrob. Proteins.

[136] Chen X., Bai H., Mo W., Zheng X., Chen H., Yin Y., Liao Y., Chen Z., Shi Q., Zuo Z. (2025). Lactic Acid Bacteria Bacteriocins: Safe and Effective Antimicrobial Agents. Int. J. Mol. Sci..

[137] Zhao S., Han J., Bie X., Lu Z., Zhang C., Lv F. (2016). Purification and Characterization of Plantaricin JLA-9: A Novel Bacteriocin against Bacillus spp. Produced by *Lactobacillus plantarum* JLA-9 from Suan-Tsai, a Traditional Chinese Fermented Cabbage. J. Agric. Food Chem..

[138] Chongyu L., Xin Y., Huilong C., Chengcheng N., Bo H., Lihua P., Xiaorong W., Yufan L., Xuekai W., Yanli L. (2025). Enterobacter spp. inhibits AFB1 production in corn silage through carbohydrates competition under rust infection. Chem. Biol. Technol. Agric..

[139] Yasmin S., Bhattacharyya S., Lahiri S. (2025). Bacteriocins in Food Safety and Food Preservation. Int. J. Biomed. Clin. Anal..

[140] Huang F., Ju Z., Hou Y., Zhao G., Yang Y., Yue B., Zhang X. (2024). Exploring advanced antimicrobial effects of *Pediococcus pentosaceus* and *Lactococcus lactis* derived from *Bufo gargarizans*: In vitro analysis and in vivo evaluation in mice. LWT.

[141] Mehl H.L., Cotty P.J. (2013). Influence of plant host species on intraspecific competition during infection by *Aspergillus flavus*. Plant Pathol..

[142] Crosier J., von Longo-Liebenstein L., Edman M., Adamczyk S., Hamberg L. (2025). Optimizing laboratory cultivation of wood-inhabiting fungi with emphasis on applied conservation. Appl. Microbiol. Biotechnol..

[143] Kong W.-L., Rui L., Ni H., Wu X.-Q. (2020). Antifungal Effects of Volatile Organic Compounds Produced by *Rahnella aquatilis* JZ-GX1 Against *Colletotrichum gloeosporioides* in *Liriodendron chinense* × *tulipifera*. Front. Microbiol..

[144] Haghshenas B., Nami Y., Haghshenas M., Abdullah N., Rosli R., Radiah D., Yari Khosroushahi A. (2015). Bioactivity characterization of Lactobacillus strains isolated from dairy products. Microbiologyopen.

[145] Gao Y., Ren H., He S., Duan S., Xing S., Li X., Huang Q. (2022). Antifungal activity of the volatile organic compounds produced by *Ceratocystis fimbriata* strains WSJK-1 and Mby. Front. Microbiol..

[146] Tenea G.N., Molina D., Cuamacas Y., Marinescu G.C., Popescu R.G. (2025). Exometabolite-Based Antimicrobial Formulations from Lactic Acid Bacteria as a Multi-Target Strategy Against Multidrug-Resistant *Escherichia coli*. Antibiotics.

[147] Jia M., Yu X., Xu K., Gu X., Harmer N.J., Zhao Y., Xiang Y., Sheng X., Li C., Du X.-D. (2024). The High-Efficiency Degradation of Multiple Mycotoxins by Lac-W Laccase in the Presence of Mediators. Toxins.

[148] Borthakur D., Sharma B.K., Duncan T. (2025). Investigating the probiotic and flavor-producing functions of Bacillus sp. FPIK1 sourced from fermented bamboo shoots of Assam. Microb. Cell Fact..

[149] Wang X., Bai Y., Huang H., Tu T., Wang Y., Wang Y., Luo H., Yao B., Su X. (2019). Degradation of Aflatoxin B1 and Zearalenone by Bacterial and Fungal Laccases in Presence of Structurally Defined Chemicals and Complex Natural Mediators. Toxins.

[150] Zhang X., Yang Y., Jiang Y., Shi L., Du H., Logrieco A.F., Moretti A., Han S., Xing F. (2026). Thermostable Oxidoreductases CotA and Prx Enable Synergistic and Peroxide-Enhanced Degradation of Aflatoxin B1. Toxins.

[151] Fruhauf S., Pühringer D., Thamhesl M., Fajtl P., Kunz-Vekiru E., Höbartner-Gussl A., Schatzmayr G., Adam G., Damborsky J., Djinovic-Carugo K. (2024). Bacterial Lactonases ZenA with Noncanonical Structural Features Hydrolyze the Mycotoxin Zearalenone. ACS Catal..

[152] Chang J.D., Wallace A.G., Foster E.E., Kim S.J. (2018). Peptidoglycan Compositional Analysis of *Enterococcus faecalis* Biofilm by Stable Isotope Labeling by Amino Acids in a Bacterial Culture. Biochemistry.

[153] Ragoubi C., Quintieri L., Greco D., Mehrez A., Maatouk I., D’Ascanio V., Landoulsi A., Avantaggiato G. (2021). Mycotoxin Removal by Lactobacillus spp. and Their Application in Animal Liquid Feed. Toxins.

[154] Cieślik A., Raczkowska J. (2025). Biocompatibility of Biomedical Materials: Reliability of Cell Viability Tests in the Context of Retinal Prostheses. Appl. Sci..

[155] Li P., Su R., Yin R., Lai D., Wang M., Liu Y., Zhou L. (2020). Detoxification of Mycotoxins through Biotransformation. Toxins.

[156] Odukoya J.O., De Saeger S., De Boevre M., Adegoke G.O., Devlieghere F., Croubels S., Antonissen G., Adebo O.A., Gbashi S., Odukoya J.O. (2023). Mycotoxin reduction and metabolite profiles of ogi produced using traditional fermentation methods. Food Hydrocoll. Health.

[157] Rämö S., Kahala M., Joutsjoki V. (2022). Aflatoxin B1 Binding by Lactic Acid Bacteria in Protein-Rich Plant Material Fermentation. Appl. Sci..

[158] Greco D., D’Ascanio V., Santovito E., Abbasciano M., Quintieri L., Techer C., Avantaggiato G. (2025). Unlocking the Potential of *Bacillus subtilis*: A Comprehensive Study on Mycotoxin Decontamination, Mechanistic Insights, and Efficacy Assessment in a Liquid Food Model. Foods.

[159] Haskard C.A., El-Nezami H.S., Kankaanpää P.E., Salminen S., Ahokas J.T. (2001). Surface Binding of Aflatoxin B 1 by Lactic Acid Bacteria. Appl. Environ. Microbiol..

[160] Shi H., Chang G., Zhang Y., Zhao Y., Wang H., Zhang J., Zhu J. (2024). Biodegradation Characteristics and Mechanism of Aflatoxin B 1 by *Bacillus amyloliquefaciens* from Enzymatic and Multiomics Perspectives. J. Agric. Food Chem..

[161] Santos J., Oliveira C., Teixeira F., Venâncio A., Silva C. (2025). Enzymatic Degradation of Ochratoxin A: The Role of Ultra-Pure Water. Foods.

[162] Sanad M.H., Farag A.B., Bassem S.A., Marzook F.A. (2022). Radioiodination of zearalenone and determination of *Lactobacillus plantarum* effect of on zearalenone organ distribution: In silico study and preclinical evaluation. Toxicol. Rep..

[163] Mischler S., André A., Freimüller Leischtfeld S., Müller N., Chetschik I., Miescher Schwenninger S. (2024). Potential of Lactic Acid Bacteria and Bacillus spp. in a Bio-Detoxification Strategy for Mycotoxin Contaminated Wheat Grains. Appl. Microbiol..

[164] Grenier B., Schwartz-Zimmermann H.E., Gruber-Dorninger C., Dohnal I., Aleschko M., Schatzmayr G., Moll W.D., Applegate T.J. (2017). Enzymatic hydrolysis of fumonisins in the gastrointestinal tract of broiler chickens. Poult. Sci..

[165] Makut M.D., Mafe A.N., Owuna J.E., Calina D., Sharifi-Rad J. (2026). Postbiotics from indigenous lactic acid bacteria: Mechanisms, biopreservation and functional food applications. J. Sci. Food Agric..

[166] Kim J.-H., Lee E.-S., Kim B.-M., Oh M.-H. (2025). Antifungal lactic acid bacteria as a biopreservative against fungal contamination in dry-cured ham. LWT.

[167] Chauhan K., Rao A. (2024). Clean-label alternatives for food preservation: An emerging trend. Heliyon.

[168] Setta M.C., Matemu A., Mbega E.R. (2020). Potential of probiotics from fermented cereal-based beverages in improving health of poor people in Africa. J. Food Sci. Technol..

[169] Nithya A., Misra S., Panigrahi C., Dalbhagat C.G., Mishra H.N. (2023). Probiotic potential of fermented foods and their role in non-communicable diseases management: An understanding through recent clinical evidences. Food Chem. Adv..

[170] Grujović M.Ž., Semedo-Lemsaddek T., Marković K.G. (2025). Application of Probiotics in Foods: A Comprehensive Review of Benefits, Challenges, and Future Perspectives. Foods.

[171] Meskini M., Rezghi Rami M., Tavakoli R., Salami M. (2026). Molecular markers in diagnostics of fungi and fungal mycotoxins: A narrative review. J. Infect. Public Health.

[172] Aboagye G., Gbolonyo-Cass S., Kortei N.K., Annan T. (2020). Microbial evaluation and some proposed good manufacturing practices of locally prepared malted corn drink (“asaana”) and *Hibiscus sabdarifa* calyxes extract (“sobolo”) beverages sold at a university cafeteria in Ghana. Sci. Afr..

[173] Tomar T., Sachdeva A., Dutta J., Al Tawaha A.R.M., Karnwal A., Malik T., Selvaraj M. (2025). Fermentation dynamics of millet beverages: Microbial interactions, nutritional enhancements, and health implications. Food Chem. X.

[174] Santos C., Raymundo A., Moreira J.B., Prista C. (2025). Exploring the Potential of Lactic Acid Bacteria Fermentation as a Clean Label Alternative for Use in Yogurt Production. Appl. Sci..

[175] Stachelska M.A., Karpiński P., Kruszewski B. (2024). Health-Promoting and Functional Properties of Fermented Milk Beverages with Probiotic Bacteria in the Prevention of Civilization Diseases. Nutrients.

[176] Lee P.Y., Leong S.Y., Oey I. (2024). The role of protein blends in plant-based milk alternative: A review through the consumer lens. Trends Food Sci. Technol..

[177] Dhiman S., Kaur S., Thakur B., Singh P., Tripathi M. (2025). Nutritional Enhancement of Plant-Based Fermented Foods: Microbial Innovations for a Sustainable Future. Fermentation.

[178] Yu J., Pedroso I.R. (2023). Mycotoxins in Cereal-Based Products and Their Impacts on the Health of Humans, Livestock Animals and Pets. Toxins.

[179] Punia Bangar S., Sharma N., Bhardwaj A., Phimolsiripol Y. (2022). Lactic acid bacteria. Qual. Assur. Saf. Crops Foods.

[180] Knez E., Kadac-Czapska K., Grembecka M. (2023). Effect of Fermentation on the Nutritional Quality of the Selected Vegetables and Legumes and Their Health Effects. Life.

[181] Wang X., Wang S., Xu J., Wu B., Hu Z., Niu H. (2024). Isolation, Characterization, and Biopreservation of *Lactobacillus brevis* DN-1 to Inhibit Mold and Remove Aflatoxin B1 in Peanut and Sunflower Cakes. Agriculture.

[182] Wacoo A.P., Mukisa I.M., Meeme R., Byakika S., Wendiro D., Sybesma W., Kort R. (2019). Probiotic Enrichment and Reduction of Aflatoxins in a Traditional African Maize-Based Fermented Food. Nutrients.

[183] Riasatian M., Mazloomi S.M., Ahmadi A., Derakhshan Z., Rajabi S. (2023). Benefits of fermented synbiotic soymilk containing *Lactobacillus acidophilus*, *Bifidobacterium lactis*, and inulin towards lead toxicity alleviation. Heliyon.

[184] Shah-Vardi M., Nazaryanpour E., Nejad-Ebrahimi S., Farzaneh M. (2022). Remediation of zearalenone mycotoxin contamination in rumen fluid by phytochemical compounds of *Zataria multiflora*. Iran. J. Vet. Res..

[185] Odukoya J.O., De Saeger S., De Boevre M., Adegoke G.O., Devlieghere F., Croubels S., Antonissen G., Odukoya J.O., Njobeh P.B. (2024). Influence of traditional dehulling on mycotoxin reduction and GC-HRTOF-MS metabolites profile of fermented maize products. Heliyon.

[186] Padmaja P.B., Selvam S.P. (2016). Determination of Antiaflatoxigenic Effect of Probiotic Strains in *Sorghum bicolour*. Biosci. Biotechnol. Res. Asia.

[187] Zain M.E. (2011). Impact of mycotoxins on humans and animals. J. Saudi Chem. Soc..

[188] Del Favero G., Hohenbichler J., Mayer R.M., Rychlik M., Marko D. (2020). Mycotoxin Altertoxin II Induces Lipid Peroxidation Connecting Mitochondrial Stress Response to NF-κB Inhibition in THP-1 Macrophages. Chem. Res. Toxicol..

[189] Hou Y.-J., Zhao Y.-Y., Xiong B., Cui X.-S., Kim N.-H., Xu Y.-X., Sun S.-C. (2013). Mycotoxin-Containing Diet Causes Oxidative Stress in the Mouse. PLoS ONE.

[190] Moulton M.J., Barish S., Ralhan I., Chang J., Goodman L.D., Harland J.G., Marcogliese P.C., Johansson J.O., Ioannou M.S., Bellen H.J. (2021). Neuronal ROS-induced glial lipid droplet formation is altered by loss of Alzheimer’s disease–associated genes. Proc. Natl. Acad. Sci. USA.

[191] Gentile F., Arcaro A., Pizzimenti S., Daga M., Cetrangolo G.P., Dianzani C., Lepore A., Graf M., Ames P.R.J., Barrera G. (2017). DNA damage by lipid peroxidation products: Implications in cancer, inflammation and autoimmunity. AIMS Genet..

[192] Wang R., Zhang Q., Chen G., Kou R., Zhang C., Wang Y., Wang J., Huang Y., Chen C. (2025). Mechanistic insights into ferroptosis and apoptosis pathways: Synergistic effects of multi-organ toxicity and transgenerational effects induced by co-exposure of epoxiconazole and aflatoxin B1 in zebrafish. J. Adv. Res..

[193] Jalili C., Abbasi A., Rahmani-Kukia N., Andarzi S., Kakebaraie S., Zamir Nasta T. (2024). The relationship between aflatoxin B1 with the induction of extrinsic/intrinsic pathways of apoptosis and the protective role of taraxasterol in TM3 leydig cell line. Ecotoxicol. Environ. Saf..

[194] Hussar P., Blagoevska K., Dovenska M., Pendovski L., Popovska-Percinic F. (2025). Immunolocalization of p53 and p21 in Kidneys Exposed to T-2 Mycotoxin. Curr. Issues Mol. Biol..

[195] Brand M.D., Orr A.L., Perevoshchikova I.V., Quinlan C.L. (2013). The role of mitochondrial function and cellular bioenergetics in ageing and disease. Br. J. Dermatol..

[196] Su L., Fang W., Zhao X., Zhu L., Gao L., Chen G. (2022). Disruption of mitochondrial redox homeostasis as a mechanism of antimony-induced reactive oxygen species and cytotoxicity. Ecotoxicol. Environ. Saf..

[197] Janik-Karpinska E., Ceremuga M., Niemcewicz M., Synowiec E., Sliwiński T., Bijak M. (2023). Mitochondrial Damage Induced by T-2 Mycotoxin on Human Skin—Fibroblast Hs68 Cell Line. Molecules.

[198] Wattanasuntorn P., Poapolathep S., Phuektes P., Alassane-Kpembi I., Fink-Gremmels J., Oswald I.P., Poapolathep A. (2025). Apoptotic Effect of Combinations of T-2, HT-2, and Diacetoxyscirpenol on Human Jurkat T Cells. Toxins.

[199] Zhao T., Jin J., Huangfu B., He X., Zhang B., Li X., Xu W., Xing F. (2024). Phlorizin Alleviates Inflammation Caused by Deoxynivalenol by Regulating the Gut Microbiome and Inhibiting the TLR4/MyD88/NF-κB Signaling Pathway in Mice. ACS Food Sci. Technol..

[200] Meng F., Zhou J., Wang H., Li Q., Zou N. (2025). Toxic effects of subchronic T-2 toxin exposure on systemic immune deficiency in developing juvenile rats. Ecotoxicol. Environ. Saf..

[201] Zhao L., Xu M., Pan X., Zhang B., Dou Q. (2021). Binding and detoxification ability of *Lactobacillus acidophilus* towards di-n-butyl phthalate: Change of MAPK pathway in Caco-2 cell model. J. Proteom..

[202] Jaglan A., Sadera G., Nagpal A., Mate P.S., Kumar S., Goel G. (2026). Protective Effects of Probiotic Mediated Digested Gliadin and 33-mer Peptide in Caco-2 Cell Cultures. Mol. Nutr. Food Res..

[203] Brunelli L., De Vitis V., Ferrari R., Minuzzo M., Fiore W., Jäger R., Taverniti V., Guglielmetti S. (2022). In vitro assessment of the probiotic properties of an industrial preparation containing *Lacticaseibacillus paracasei* in the context of athlete health. Front. Pharmacol..

[204] Demirhan H.K., Omer Oglou E., Aksoy Z.B., Kiran F. (2025). Evaluation of the anti-inflammatory, antioxidant and regenerative effects of microbiota-derived postbiotics in human periodontal ligament mesenchymal stromal cells. Clin. Oral Investig..

[205] Aydemir Atasever M., Güler İnce M.B., Alkan Polat B., Özlü H., Atasever M. (2025). Aflatoxin B1 levels, dietary exposure and cancer risk assessment in sesame and nut-based foods in Türkiye. Mycotoxin Res..

[206] Rafai S., Moreno A., Cimbalo A., Vila-Donat P., Manyes L., Meca G. (2025). In Vitro Evaluation of Aflatoxin B1 Detoxification by Lactobacillus, Pediococcus, and Bacillus Strains. Toxins.

[207] Moradkhani F., Sekhavatizadeh S.S., Marhamatizadeh M.H., Ghasemi M. (2025). Biodetoxification of Aflatoxin M1 in Artificially Contaminated Fermented Milk, Fermented Dairy Drink and Yogurt Using *Lactobacillus acidophilus*, *Lactobacillus plantarum*, *Lactobacillus reuteri*, and *Lactobacillus rhamnosus* and Its Effects on Physicochemical Properties. Food Sci. Nutr..

[208] Iram W., Anjum T., Iqbal M., Ghaffar A., Abbas M. (2016). Structural Elucidation and Toxicity Assessment of Degraded Products of Aflatoxin B1 and B2 by Aqueous Extracts of *Trachyspermum ammi*. Front. Microbiol..

[209] Sanaldi K., Coban A.Y. (2023). Detoxification of aflatoxin M1 in different milk types using probiotics. An. Acad. Bras. Cienc..

[210] Albulaihed Y., Adnan M., Jamal A., Snoussi M., Patel K., Patel M. (2023). Optimization of laccase from *Stenotrophomonas maltophilia* E1 by submerge fermentation using coconut husk with its detoxification and biodecolorization ability of synthetic dyes. Bioresour. Bioprocess..

[211] Lara-Moreno A., Rodriguez-Morillo B., Madrid F., Martin-Sanchez P.M., Villaverde J., Mejías C., Alonso E., Santos J.L., Morillo E. (2026). Enhanced Diclofenac Biodegradation by Bacterial Strains and a Microbial Consortium from Activated Sludge: Toxicity Assessment and Insights into Microbial Community Dynamics. J. Xenobiotics.

[212] Ajagbe D., Krzmarzick M., Fathepure B. (2026). Bioremediation of Produced Water by a Polyextremophilic, Heavy-Metal-Resistant Modicisalibacter sp. Strain Wilcox. ACS ES&T Water.

[213] Zhang H., Yu Y., Yang J., Tang Y. (2025). A novel *Bacillus paramycoides* HS-1 capable of degrading aflatoxin B1 and its key enzymatic mechanisms. Food Biosci..

[214] Elsaadany K., Badr A.N., Ibrahim A., Madian R., Kan J., Du M., Awad S. (2025). Safety, probiotic potential, and mycotoxin-reduction efficacy of *Limosilactobacillus fermentum* isolated from traditional dairy products. Appl. Food Res..

[215] Islam S., Biswas S., Jabin T., Moniruzzaman M., Biswas J., Uddin M.S., Akhtar-E-Ekram M., Elgorban A.M., Ghodake G., Syed A. (2023). Probiotic potential of *Lactobacillus plantarum* DMR14 for preserving and extending shelf life of fruits and fruit juice. Heliyon.

[216] Al-Kharousi Z.S. (2025). Highlighting Lactic Acid Bacteria in Beverages: Diversity, Fermentation, Challenges, and Future Perspectives. Foods.

[217] Ashaolu T.J., Varga L., Greff B. (2025). Nutritional and functional aspects of European cereal-based fermented foods and beverages. Food Res. Int..

[218] Aguirre-Garcia Y.L., Nery-Flores S.D., Campos-Muzquiz L.G., Flores-Gallegos A.C., Palomo-Ligas L., Ascacio-Valdés J.A., Sepúlveda-Torres L., Rodríguez-Herrera R. (2024). Lactic Acid Fermentation in the Food Industry and Bio-Preservation of Food. Fermentation.

[219] Pop O.L., Suharoschi R., Gabbianelli R. (2022). Biodetoxification and Protective Properties of Probiotics. Microorganisms.

[220] Sionek B., Szydłowska A., Jaworska D., Kołożyn-Krajewska D. (2025). Benefits of Probiotics—Biodetoxification. Appl. Sci..

[221] Xiang H., Sun-Waterhouse D., Waterhouse G.I.N., Cui C., Ruan Z. (2019). Fermentation-enabled wellness foods: A fresh perspective. Food Sci. Hum. Wellness.

[222] Mafe A.N., Büsselberg D. (2025). Probiotics and Postbiotics for Green Control of Foodborne Pathogens: Intelligent Detection and Biopreservation Strategies for Safer Foods. Foods.

[223] Bereda G. (2025). Toxicological impacts and mitigation strategies of food contaminants: A global perspective and comprehensive narrative review. Curr. Res. Toxicol..

[224] Adeniji O.O., Akinmoladun O.F., Njobeh P.B. (2025). Microbial interventions for aflatoxin control in food systems: A 25-year global bibliometric analysis (2000–2024) with implications for food security and smart agriculture. Food Saf. Risk.

[225] Siddiqui S.A., Erol Z., Rugji J., Taşçı F., Kahraman H.A., Toppi V., Musa L., Di Giacinto G., Bahmid N.A., Mehdizadeh M. (2023). An overview of fermentation in the food industry—Looking back from a new perspective. Bioresour. Bioprocess..

[226] Kibugu J., Munga L., Mburu D., Maloba F., Auma J.E., Grace D., Lindahl J.F. (2024). Dietary Mycotoxins: An Overview on Toxicokinetics, Toxicodynamics, Toxicity, Epidemiology, Detection, and Their Mitigation with Special Emphasis on Aflatoxicosis in Humans and Animals. Toxins.

[227] Masquelier J., Tangni E.K., Becker P., Sanders J., Laporte J., Mertens B. (2025). Validation of an LC-MS Method for Quantification of Mycotoxins and Characterization of Fungal Strains Occurring in Food and Feed. Chemosensors.

[228] Dasí-Navarro N., Lombardi S., Vila-Donat P., Llop S., Vioque J., Soler-Blasco R., Esplugues A., Manyes L., Lozano M. (2025). Metabolomic Profiling of Human Urine Related to Mycotoxin Exposure. Toxins.

[229] Liu L., Xie M., Wei D. (2022). Biological Detoxification of Mycotoxins: Current Status and Future Advances. Int. J. Mol. Sci..

[230] Vijayaram S., Vivekanandan K.E., Kandasamy S., Razafindralambo H., Ringø E., Sun Y.-Z., Kaliyannan G. (2025). Probiotics: A multifaceted approach to health promotion-from disease prevention to food enrichment and delivery systems. AIMS Microbiol..

[231] Churin A.A., Sokolyanskaya L.O., Lukina A.P., Karnachuk O.V. (2026). Current Concepts in Probiotic Safety and Efficacy. Nutrients.

[232] Osselaere A., Devreese M., Watteyn A., Vandenbroucke V., Goossens J., Hautekiet V., Eeckhout M., De Saeger S., De Baere S., De Backer P. (2012). Efficacy and safety testing of mycotoxin-detoxifying agents in broilers following the European Food Safety Authority guidelines. Poult. Sci..

[233] Greco D., D’Ascanio V., Abbasciano M., Treglia A., Avantaggiato G. (2026). In Vitro Efficacy Assessment of Mycotoxin-Detoxifying Agents Against Emerging Mycotoxins. Agriculture.

[234] Li X., Shen X., Jiang W., Xi Y., Li S. (2024). Comprehensive review of emerging contaminants: Detection technologies, environmental impact, and management strategies. Ecotoxicol. Environ. Saf..

[235] Chen R., Tu H., Chen T. (2022). Potential Application of Living Microorganisms in the Detoxification of Heavy Metals. Foods.

[236] Koutsoumanis K., Allende A., Alvarez-Ordóñez A., Bolton D., Bover-Cid S., Chemaly M., Davies R., De Cesare A., Hilbert F., Lindqvist R. (2019). Whole genome sequencing and metagenomics for outbreak investigation, source attribution and risk assessment of food-borne microorganisms. EFSA J..

[237] Kumar A., Das S., Ali S., Jaiswal S.G., Rabbani A., Rahman S.M.E., Chelliah R., Oh D.-H., Liu S., Wei S. (2025). Mechanisms, applications and challenges of natural antimicrobials in food system. Food Biosci..

[238] Huys G., Botteldoorn N., Delvigne F., De Vuyst L., Heyndrickx M., Pot B., Dubois J., Daube G. (2013). Microbial characterization of probiotics–Advisory report of the Working Group “8651 Probiotics” of the Belgian Superior Health Council (SHC). Mol. Nutr. Food Res..

[239] Roe A.L., Boyte M.-E., Elkins C.A., Goldman V.S., Heimbach J., Madden E., Oketch-Rabah H., Sanders M.E., Sirois J., Smith A. (2022). Considerations for determining safety of probiotics: A USP perspective. Regul. Toxicol. Pharmacol..

[240] Kim H., Haque M.A., Razzak M.A., Jang M.J., Song S., Ku S. (2026). Probiotic Development Strategy Centered on Stability and Regulatory Considerations. Compr. Rev. Food Sci. Food Saf..

